# Selenium Supplementation Restores Innate and Humoral Immune Responses in Footrot-Affected Sheep

**DOI:** 10.1371/journal.pone.0082572

**Published:** 2013-12-05

**Authors:** Jean A. Hall, William R. Vorachek, Whitney C. Stewart, M. Elena Gorman, Wayne D. Mosher, Gene J. Pirelli, Gerd Bobe

**Affiliations:** 1 Department of Biomedical Sciences, College of Veterinary Medicine, Oregon State University, Corvallis, Oregon, United States of America; 2 Department of Animal and Rangeland Sciences, College of Agriculture, Oregon State University, Corvallis, Oregon, United States of America; 3 Current Address: Department of Animal and Range Sciences, New Mexico State University, Las Cruces, New Mexico, United States of America; 4 Linus Pauling Institute, Oregon State University, Corvallis, Oregon, United States of America; Indian Institute of Science, India

## Abstract

Dietary selenium (Se) alters whole-blood Se concentrations in sheep, dependent upon Se source and dosage administered, but little is known about effects on immune function. We used footrot (FR) as a disease model to test the effects of supranutritional Se supplementation on immune function. To determine the effect of Se-source (organic Se-yeast, inorganic Na-selenite or Na-selenate) and Se-dosage (1, 3, 5 times FDA-permitted level) on FR severity, 120 ewes with and 120 ewes without FR were drenched weekly for 62 weeks with different Se sources and dosages (30 ewes/treatment group). Innate immunity was evaluated after 62 weeks of supplementation by measuring neutrophil bacterial killing ability. Adaptive immune function was evaluated by immunizing sheep with keyhole limpet hemocyanin (KLH). The antibody titer and delayed-type hypersensitivity skin test to KLH were used to assess humoral immunity and cell-mediated immunity, respectively. At baseline, FR-affected ewes had lower whole-blood and serum-Se concentrations; this difference was not observed after Se supplementation. Se supplementation increased neutrophil bacterial killing percentages in FR-affected sheep to percentages observed in supplemented and non-supplemented healthy sheep. Similarly, Se supplementation increased KLH antibody titers in FR-affected sheep to titers observed in healthy sheep. FR-affected sheep demonstrated suppressed cell-mediated immunity at 24 hours after intradermal KLH challenge, although there was no improvement with Se supplementation. We did not consistently prevent nor improve recovery from FR over the 62 week Se-treatment period. In conclusion, Se supplementation does not prevent FR, but does restore innate and humoral immune functions negatively affected by FR.

## Introduction

Dietary selenium (Se) alters whole-blood (WB) Se concentrations in sheep, depending upon the chemical source and dosage administered [1]–[5]. Less is known about how different chemical forms of Se (inorganic Na-selenate or Na-selenite, and organic Se-yeast) at comparative dosages alter immune functions. In domestic animals, including sheep, Se deficiency results in immunosuppression. Specifically, Se deficiency decreases resistance to bacterial and viral infections, and decreases neutrophil function, antibody production, proliferation of T and B lymphocytes in response to mitogens, and cytodestruction by T lymphocytes and natural killer cells (reviewed in [6]–[10]). The effect of supranutritional Se supplementation on specific immune functions has not been well studied. We hypothesize that the amount of Se required for optimum health is higher than the amount required for prevention of nutritional myodegeneration [11].

Current Food and Drug Administration (FDA) regulations limit the amount of dietary Se supplementation, regardless of chemical source, to 0.3 mg/kg (as fed), or 0.7 mg per sheep per day [12]. Concentrations that exceed 0.3 mg/kg but that are less than the maximum tolerable level (5 mg/kg of diet, as fed) are referred to as supranutritional. There is interest in supranutritional supplementation relative to health, performance, and disease prevention in animals and humans [13]–[15]. We recently reported that supranutritional Se supplementation improved response to vaccination with a J-5 *Escherichia coli* bacterin in adult beef cows [16]. Furthermore, we reported that supranutritional Se supplementation of ewes improves growth and survival of their offspring [17], which may be due in part to greater colostral IgG concentrations and greater calculated amounts of IgG transferred to their lambs [18], suggesting that supranutritional Se supplementation may enhance passive immunity.

A suitable model in sheep to test the effects of supranutritional Se supplementation on immune function is footrot (FR), an endemic bacterial disease of sheep feet that results in lameness and large production loses for the industry. Footrot is caused by infection with *Dichelobacter nodosus*, a gram negative, anaerobic and fastidious bacterium, in association with other bacteria, particularly *Fusobacterium necrophorum* [19]–[21]. If the interdigital skin of the foot is damaged or wet for prolonged periods of time, it may become infected by the ubiquitous soil and fecal bacterium *F. necrophorum*. *F. necrophorum* causes interdigital dermatitis and produces toxins that cause necrosis of the superficial layer of the skin allowing co-infection with other bacteria such as *D. nodosus*. *D. nodosus* contains surface fimbriae and stable extracellular proteases that allow it to colonize the interdigital epithelial tissue, digesting the living dermis, and feeding on collagen [22], [23]. A foul smell is associated with the accumulation of grey pasty exudate between the dermis and epidermal horn, and ultimately separation of the hoof horn from the underlying dermis occurs [23]. A strict culling program during the hot, dry summer months (non-transmission period) has proven successful in eliminating FR in flocks in Western Australia [24]. However, this protocol is unfeasible in countries with cool, wet climates and widespread infection in flocks [23]. Instead, management programs to control rather than eliminate infection are more commonly employed. Strategies include parenteral antibiotic treatment, topical antibacterial sprays, trimming of horn hoof, vaccination, low stocking density, and genetic selection for sheep breeds less susceptible to FR [23], [25]. We previously documented in a small-scale study that WB-Se concentrations are lower in FR-affected compared with healthy sheep, and that supranutritional Se-supplementation (inorganic sodium selenite administered parenterally once monthly) hastens FR recovery [26].

The role of the immune response and Se supplementation in the pathogenesis or recovery from FR is unclear. Adaptive immunity, including humoral and cell mediated immunity (CMI), likely play a role in protection against FR [27], [28], yet infected or vaccinated sheep do not develop long-term immunity and may become re-infected over time [23], [29], [30]. Heritability of resistance to FR may be related to a specific range of MHC II haplotype that is required to generate a sufficient immune response to *D. nodosus* [28]. We recently showed that supranutritional Se-yeast supplementation in ewes increased WB-neutrophil expression of genes involved in innate immunity, including reversing those impacted by FR [31].

The objective of the current study was to determine the effects of Se-source (inorganic Na-selenate or Na-selenite versus organic Se-yeast) and Se-dosage (0, 1, 3, or 5 times the FDA-allowed Se supplementation rate) on immune function. We hypothesized that both arms of the immune response, i.e., innate immunity of neutrophils and adaptive immunity (both humoral and cell mediated immunity), would be enhanced by supplementation with supranutritional levels of Se, with a greater benefit from Se-yeast. Innate immunity was evaluated by measuring the bacterial killing ability of isolated neutrophils. Humoral immunity was evaluated by measuring an antibody titer response to a novel protein challenge (KLH, keyhole limpet hemocyanin). Cell mediated immunity was evaluated using a delayed hypersensitivity test (DTH) to KLH. Finally, we monitored the incidence and severity of FR across time in sheep receiving varying dosages of organic versus inorganic Se.

## Materials and Methods

### Animals, Experimental Design, and Selenium Treatments

**Figure 1:**
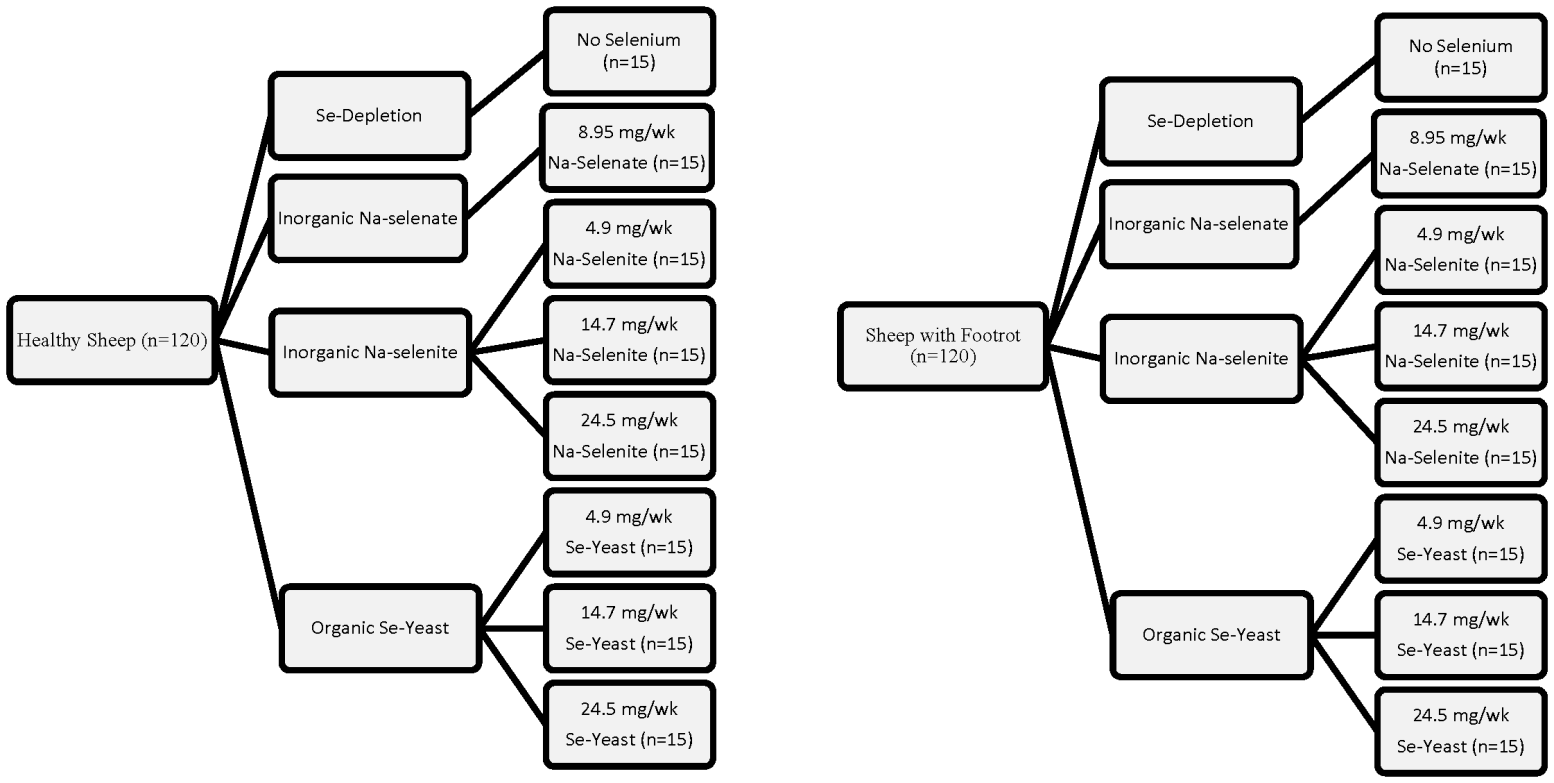
Study design. Mature breeding ewes were randomly assigned to 8 treatment groups (n = 30 each; 15 healthy and 15 foot rot affected) for 62 wk based on Se source (no Se, inorganice Na-selenate, inorganic Na-selenite, and organic Se-yeast) and Se supplementation rate (0, 4.9, 14.7, and 24.5 mg Se/wk; Na-selenatate only at 8.95 mg/wk Se/wk)**.**

Experimental procedures used in this study were approved by the Institutional Animal Care and Use Committees of Oregon State University (Permit Number: 3778) and have been described in detail previously [1]. As shown in **Figure 1**, mature breeding ewes that had been randomly assigned to 8 treatment groups (n = 30 each; 15 healthy and 15 foot rot affected) based on Se supplementation rate and source were used: one group received water (no Se supplement group), one group received Na-selenate (8.95 mg Se/wk), 3 groups received Na-selenite (RETORTE Ulrich Scharrer GmbH, Röthenbach, Germany) at 4.9 (maximum FDA-allowed level), 14.7 (3 times maximum FDA-allowed level), or 24.5 mg Se/wk (5 times maximum FDA-allowed level), and 3 groups received Se-yeast (Prince Se Yeast 2000, Prince Agri Products Inc., Quincy, IL) at 4.9, 14.7, or 24.5 mg Se/wk. Sodium-selenate (Na_2_SeO_4_) was 418 g/kg Se or 41.8% Se, and Na-selenite (Na_2_SeO_3_) was 456 g/kg Se or 45.6% Se. The organic Se source had a guaranteed analysis of 2 g/kg of organically bound Se with 78% being selenomethionine (SeMet).

The treatment period started approximately 2 wk before breeding and lasted for 62.5 wk. All dosages were below the maximum tolerable level (5 mg/kg as fed, which is 16.7 times the FDA-allowed Se supplementation rate) for small ruminants [32]. Treatment groups were stratified for age of ewe and FR severity (scale 0 to V; with no FR  =  0 being the lowest category). Ewes were from three genotypes (Polypay, Suffolk, and Suffolk by Polypay cross), and ranged in age and body weight (BW) from 2 to 6 yr, and 51 to 93 kg, respectively. The three genotypes were not completely balanced across treatments. The experiments were conducted at the Oregon State University Sheep Center, Corvallis, OR.

Ewe treatments were administered individually once per wk by oral drench (with the calculated weekly amount of Se supplement being equal to the summed daily intake). The weekly Se dose (4.9, 8.95, 14.7, or 24.5 mg Se/wk per ewe) was suspended in water (11, 11, 30, and 48 mL for the 4.9, 8.95, 14.7, or 24.5 mg Se solutions, respectively). Stock solutions were made up fresh each wk and administered with a dose syringe. To ensure a homogeneous dosage, stock solutions were stirred each time prior to being drawn into the dose syringe. The no-Se treatment group received 11 mL water.

Individual treatment aliquots were submitted for Se analysis to the Center for Nutrition, Diagnostic Center for Population and Animal Health, Michigan State University (East Lansing, MI), and Se was quantified according to previously described methods [1]. The 4.9, 14.7, and 24.5 mg weekly dosages of Na-selenite (4.9, 14.9, and 24.6 mg Se, respectively), and the weekly dosages of organic Se-yeast supplements (4.8, 14.4, and 24.0 mg of organically bound Se-yeast, respectively) were found to be within expected analytical variance of their targeted concentrations. The dosage of Na-selenate administered was determined to be 8.95 mg Se/wk per ewe. Concentrations of Se in the pasture forage from the sheep center pastures ranged from 0.12 to 0.14 mg/kg dry matter [1]. The Se concentrations of the grass hay, alfalfa hay, alfalfa pellets, whole corn, and custom-made mineral supplement (Oregon State University Sheep Mineral Premix, Wilbur-Ellis Company, Clackamas, OR) were 0.02, 0.05, 0.06, 0.01, and 0.44 mg Se/kg dry matter, respectively. Assuming pasture dry matter intake of 2% of BW, ewes would consume between 0.12 and 0.26 mg Se/d. For an average mineral intake of 8 g/d, an additional 3 µg of Se would be consumed. Other feed ingredients would contribute less than 20 µg Se/d. Thus, the majority of Se intake (4.9, 14.7, or 24.5 mg Se/wk) was provided by the oral Se drench [1].

Ewes were fed on pasture, except for a 3-mo period around lambing when ewes were housed in the barn. Ewes on pasture were supplemented with grass hay and later with alfalfa hay when grass was scarce. In the barn, sheep were fed alfalfa hay and shelled corn, except for 2 d in the lambing jug when ewes were fed alfalfa pellets. Ewe feed sources and management details have been previously described [1]. Sheep were fed to meet or exceed National Research Council [32] recommendations.

### Foot Scores

At 0, 20, 28, 40, and 60 wk of Se supplementation, each sheep foot was examined, trimmed, and scored for FR using a scale of 0 to 4. This scoring system was based on pathologically defined criteria [33]. Definitions for the scores per foot are as follows: 0: normal hoof, no evidence of FR; 1: interdigital dermatitis, presence of heat, and characteristic FR odor; 2: initial underrunning of the hoof wall between the toes; 3: underrunning of the sole; and 4: extensive underrunning of the sole and lateral walls of the hoof. The same person assigned scores (blinded) at each time point for consistency of evaluations.

Based on the FR scores, sheep were classified into the following FR severity categories: Category 0: normal hoofs, no evidence of FR; Category I: single foot affected and FR score of 1 or 2; Category II: single foot affected and FR score of 3 or 4; Category III: at least 2 feet affected and at least one foot with low FR score of 1 or 2; Category IV: at least 2 feet affected with FR scores equal or greater than 3; and Category V: at least 3 feet affected, FR score greater than 3 in at least one foot, and FR score equal to or greater than 2 in other feet. For each treatment group (n = 30) sheep were randomly assigned as follows: Category 0 (n = 15), Category I (n = 4), Category II (n = 5), Category III (n = 3), Category IV (n = 2), and Category V (n = 1).

Sheep used in this study had naturally acquired FR. The FR control program [34] in this flock consisted of a walkthrough footbath containing 10% zinc sulfate solution (used monthly or less often). A FR vaccination program was not used. The guidelines for administration of parenteral oxytetracycline antibiotic (Liquamycin LA-200; Pfizer Animal Health, Exton, PA) at the 28- and 40-wk foot trimming sessions were if one foot had a score of 4, or one foot had a score of 3 and a second foot had a score of 2 or greater, or if all 4 feet had FR, then 20 mg/kg oxytetracycline was administered subcutaneously.

### Blood Collection and Se Analysis

Jugular venous blood was collected from ewes monthly. For this study, blood samples from 0, 14, 27, 40, and 54 wk of Se supplementation were used. Whole blood was collected into evacuated tubes without EDTA (10 mL; Becton Dickinson, Franklin Lakes, NJ). Tubes were centrifuged at 850 × *g* for 10 min and serum was harvested, centrifuged again at 16,300 × *g* for 1 min in a microcentrifuge to remove remaining red blood cells (RBC), and transferred to 2.0 mL screw cap self-standing microcentrifuge tubes and stored at −20°C for a serum-Se assay. Jugular venous blood was collected into evacuated tubes with EDTA (2 mL; final EDTA concentration 2 g/L; Becton Dickinson) and stored on ice until it could be frozen at −20°C for a WB-Se assay. Selenium concentrations in WB and serum were determined by a commercial laboratory (Center of Nutrition, Diagnostic Center for Population and Animal Health, Michigan State University) using ionized coupled plasma mass spectrometry (ICP-MS) method with modifications as previously described [1]. Finally, jugular venous blood was collected at 14 months into EDTA tubes (10 mL; final EDTA concentration 2 g/L, Becton Dickinson) and transported on ice to the lab for subsequent isolation of neutrophils.

### Isolation of peripheral blood neutrophils

Neutrophils were isolated within 4 h of collection, using a Percoll (Sigma-Aldrich, St. Louis, MO) gradient technique, then resuspended in 1× Hank’s balanced saline solution (HBSS; Life Technologies, Grand Island, NY) plus 0.5% fetal bovine serum (FBS; Life Technologies). Cells were counted using a Coulter counter (Beckman Coulter, Indianapolis, IN) to determine cell concentration. Briefly, 10 mL of anticoagulated blood was transferred into 50-mL tubes (Thermo Fisher Scientific, Waltham, MA) and centrifuged at 1000×*g* in a TJ-6 swinging bucket centrifuge (Beckman Coulter) for 20 min at 22 °C. The plasma, buffy coat, and one third of the RBC pack from each tube were aseptically removed. The remaining RBC pack and leukocytes were mixed with 34-mL ice-cold phosphate buffered saline (PBS; Life Technologies) and layered onto 10 mL of freshly prepared 1.084 g/mL Percoll. Tubes were centrifuged at 400×*g* for 40 min at 22 °C. After centrifugation, RBC and neutrophils pelleted at the bottom of the tube. The mononuclear cell band remained at the sample/medium interface, and was aspirated and discarded.

Neutrophils were isolated from RBC by lysing the RBC with 24-mL ice-cold hypotonic lysis buffer (10.56 mM Na_2_HPO_4_, 2.67 mM NaH_2_PO_4_, pH 7.3) for 90 s, and then isotonicity was restored by adding 12-mL ice-cold hypertonic restore buffer (10.56 mM Na_2_HPO_4_, 2.67 mM NaH_2_PO_4_, 0.43 M NaCl, pH 7.3) to stop lysis. Neutrophils were pelleted by centrifugation of tubes at 800×*g* for 5 min at 22 °C in a TJ-6 centrifuge. The lysis solution was decanted, and the neutrophils were resuspended and washed twice more with 1× HBSS plus 0.5% FBS. The neutrophils were then resuspended in 0.25 mL of 1× HBSS with 0.5% FBS and stored on ice until needed. A 20-µL aliquot of the cell suspension was used to determine cell concentration using a Coulter counter (Beckman). Another 5-µL aliquot was used to assess purity of neutrophil preparations (differential cell count) by microscopic examination after Wright-Giemsa staining (96±1% neutrophils; mean ± SEM).

### Neutrophil bacterial killing of Lactococcus lactis or Escherichia coli

To evaluate innate immune function, we measured neutrophil bacterial killing after 14 months of Se supplementation using a cell proliferation assay (CellTiter 96® Non-Radioactive Cell Proliferation Assay; Promega Corp, Madison, WI). In this assay, living bacteria convert the tetrazolium component of the dye solution into a formazan product during the 4-h incubation. A solution is added to wells to solubilize the formazan product and absorbance at 570 nm is recorded using a 96-well plate reader. In short, *Escherichia coli* (*E. coli*) were grown overnight to stationary phase in 5-mL Luria Broth media (prepared using ingredients from Thermo Fisher Scientific) after which the bacteria were put on ice. The bacteria were washed twice with 1× HBSS (centrifuged at 4 °C in a TJ-6 centrifuge at 1300×*g* for 10 min), and then resuspended in 1× HBSS to approximately 2×10^5^ colony forming units (CFU)/μL and stored on ice until needed. Next 1.2×10^6^ CFU of *E. coli* were added to all experimental wells of a 96-well flat bottom plate (Corning Inc., Corning, NY). Neutrophils (1.2×10^5^ cells) from each calf were seeded into triplicate wells containing bacteria, and 1× HBSS added such that the total volume was 50 µL. The multiplicity of infection (MOI) was 10 bacteria to 1 neutrophil. A standard curve was prepared from serial dilutions of bacteria in 1× HBSS (2.4×10^6^ to 7.5×10^4^ CFU); these bacterial dilutions were seeded into triplicate wells without neutrophils. Three wells without bacteria or neutrophils were used for background controls. The 96-well plate was covered and incubated at 37 °C in a 5% CO_2_ humidified chamber for 2 h for neutrophil bacterial killing. After incubation, 50 mL of a 0.2% saponin solution (Sigma-Aldrich) was added to each well to lyse remaining neutrophils, and the plate was incubated at 37°C for 1 h in a 5% CO_2_ humidified chamber. After incubation was complete, 15 µL of dye solution was added to each well and the plate was incubated at 37°C for 4 h in a 5% CO_2_ humidified chamber. Then 100 µL of solubilization solution/stop mix was added to each well and the plate was incubated overnight at ambient temperature in the dark. The 96-well plate was then scanned at 570 nM using a BioRad plate reader (BioRad Inc., Hercules, CA). Data was analyzed using Excel (Microsoft Inc., Bellevue, WA) to determine the number of live bacteria in each well based on the bacteria standard curve. Final data are expressed as % bacteria killed.

### Delayed-type hypersensitivity (DTH) skin test with keyhole limpet hemocyanin (KLH)

Adaptive immune function was evaluated after 12 months of Se supplementation by immunizing all sheep twice 2-weeks apart with 0.5 mL of KLH (500 µg of KLH emulsified in 1.0 mg of T1501 adjuvant for a total volume of 0.5 mL; administered intramuscularly) as previously described [35]. Three weeks after the second KLH immunization, intradermal skin testing was performed. Swelling and induration following an intradermal challenge are measured in the DTH test to assess the CMI response by T cells. Individual disposable tuberculin syringes were filled with heat aggregated KLH and a 25-gauge needle was used to inject 0.05 mL of KLH intradermally on the ear tip. The 0.05-mL dose of heat-aggregated KLH consisted of approximately 3 mg of KLH, and was prepared as described previously [35]. Measurements of wheal diameter and ear thickness were made at 30 min (to test innate immune function) and at 24, 48, 72, and 96 h for the DTH test. Reactions were recorded according to the diameter of induration and erythema. The test was administered by the same person to all sheep. No chemical restraint was used.

### KLH antibody titer

The KLH antibody titer was also used to assess adaptive immune function. The humoral immune response was evaluated after 12 months of Se supplementation by comparing the KLH antibody titer before vaccination with KLH (after 9 months of Se supplementation and 2 months before vaccination) to the titer obtained 3.5 weeks after the second KLH immunization.

Serum was assayed for KLH antibody titer by an indirect ELISA. Briefly, 96-well microtiter plates (Costar, Cambridge, MA, USA) were coated with 5 µg/mL of KLH (100 µL/well; Sigma Chemical Co., St. Louis, MO, USA) in a 0.1 M sodium bicarbonate buffer, sealed to prevent evaporation, and incubated at 4°C overnight. The next day the coating solution was removed and 100 µL of StabilCoat (SurModics Inc., Eden Praire, MN, USA) was added to each well to block nonspecific binding sites and plates were incubated for 30 min at room temperature. After incubation, the StabilCoat was removed; plates were resealed and stored at 4°C until used. Serum samples were serially diluted (1∶100 to 1∶1,024,000) in 0.05 M PBS with 0.05% Tween-20 (T-PBS; pH 8.0). Each dilution was added to the plate in duplicate, and incubated for 30 min at room temperature. Positive and negative control serums were included on each plate. After incubation, plates were washed eight times with T-PBS and then 100 µL of horseradish peroxidase conjugated to rec-Protein G (Zymed Laboratories Inc., San Francisco, CA, USA) was added to each well at a previously determined dilution (1∶20,000). Rec-Protein G binds to IgG immunoglobulin through their Fc regions and was used instead of an anti-species conjugate as it resulted in equivalent results with a stronger signal and less background. After another 30 min incubation at room temperature, plates were again washed 5 times with T-PBS and 100 µL of 3, 3’, 5, 5’-tetramethyl benzidine (TMB; Sigma, St Louis, MO) was added to each well. Plates were read at 655 nm until an absorbance of 1.000 O.D. was reached in the least dilute standard well. The TMB reaction was then stopped by adding 100 µL of 1M H_2_SO_4_ and the plate was read at 450 nm.

For standards, we used pooled sheep serum at each time point before and after immunization. The change in titer from pre-immunization to 3.5 weeks after the second immunization was determined using a fold multiple calculation based on the pooled sheep serum standards. Ten µL of serum from each ewe before immunization were pooled into a single tube and mixed, and aliquots of this pooled serum were placed into screw cap tubes for later use as a pre immunization-pooled standard. The same procedure was followed for the post-immunization-pooled standard. On every ELISA plate, a duplicate set of eight dilutions from the pooled standard was assayed along with a set of serum dilutions from each individual ewe. The dilutions of pooled serum provided a log linear standard curve that was used to normalize all ELISA plates. A log linear curve was then generated for each individual ewe sera. A linear equation was derived in Excel from the log linear portion of each curve and the quantity of serum that would have been needed to achieve an absorbance of 0.40 at 450 nm was calculated. The amount of serum from each individual ewe that would have been needed to achieve an absorbance of 0.40 was compared to the amount of pooled serum standard needed to achieve an absorbance of 0.40 and expressed as an inverse fold multiple. Values are inverse fold multiples of log transformed raw data.

### Statistical analysis

Statistical analyses were performed using SAS, version 9.2 (SAS, Inc., Cary, NC, USA) software. For evaluating the effect of Se supplementation on WB-Se and serum-Se concentration, data were analyzed in PROC GLM. Fixed effects in the model were Se-source and dosage (no-Se, 8.95 mg Na-selenate/wk, 4.9 mg Na-selenite/wk, 14.7 mg Na-selenite/wk, 24.5 mg Na-selenite/wk, 4.9 mg Se-yeast/wk, 14.7 mg Se-yeast/wk, and 24.5 mg Se-yeast/wk), ewe FR status at the time of blood collection (no FR, FR), ewe breed (Polypay, Suffolk, or Suffolk x Polypay; the Suffolk and Suffolk x Polypay ewes were combined into one group because animal numbers for both groups were small and phenotypically the crossbreds more closely resembled the Suffolks in size; the reference group was Polypay ewes), ewe age at lambing (<5, 5, >5 yr), and the interaction between FR status at the time of blood collection and Se-source and dosage.

For evaluating the effect of Se source and dosage on FR prevalence and severity, data were analyzed in PROC GLIMMIX assuming a binomial distribution for prevalence of FR (% ewes with FR or % ewes with FR ≤ I) and assuming a negative binomial distribution for FR severity (0 to V). Fixed effects in the model were the same as for blood Se concentrations. We conducted a stratified analysis by FR status to evaluate whether Se supplementation improved FR prevalence and severity in ewes affected with FR. The effect of Se source and dosage on response to oxytetracycline treatment at 28 wk and 40 wk of supplementation (yes  =  FR severity 0 or I, no  =  FR severity ≥ II) was evaluated utilizing the same statistical model as for FR prevalence.

Indicators of innate and adaptive immunity were analyzed using PROC GLM (neutrophil bacterial killing assay, 30-min skin test response to intradermal KLH injection, and KLH antibody titers) for single measures in time and PROC MIXED (DTH skin test responses) for repeated measures in time within the same animals. Fixed effects in the model were the same as previously described. For DTH, additional fixed effects in the model were time of measurement (24, 48, 72, and 96 hr after KLH challenge) and the interactions of Se source and dosage by time, FR status by time, and Se source by FR status by time. A completely unrestricted variance-covariance structure was used to account for repeated measures taken on individual ewes across time. To obtain the correct degrees of freedom, the KENWARDROGER option was invoked. The KENWARDROGER option consists of the Satterthwaite adjustment for degrees of freedom with a Kenward-Roger adjustment on standard errors, which can be used for repeated measures studies. Antibody titers for KLH were normalized by log 2 transformation.

The effect of Se-source and dosage were evaluated using preplanned comparisons. The effect of Se supplementation was evaluated by comparing the estimated values of the no Se group with the average of the estimated values of the seven Se-supplemented ewe groups. The effect of Se source was evaluated by comparing the three Se sources (no Se, Na-selenate, Na-selenite, and Se-yeast), for which the average of the estimated values of the three Na-selenite and three Se-yeast groups were used. The effect of Se dosage was evaluated by comparing the estimated values of the three Na-selenite and three Se-yeast dosage groups (4.5, 14.7, 24.5 mg/wk). The effect of supranutritional Se dosage was evaluated by comparing the average of the estimated values of the two higher dosages (14.7 and 24.5 mg/kg) with that of the maximal FDA-allowed dosage (4.5 mg/wk). Data are reported as least square means ± SEM except for FR lesions. All statistical tests were two-sided. Statistical significance was declared at P ≤ 0.05 and tendencies at 0.05 <P ≤ 0.10.

## Results

### Blood Se concentrations and ewe health

**Figure 2:**
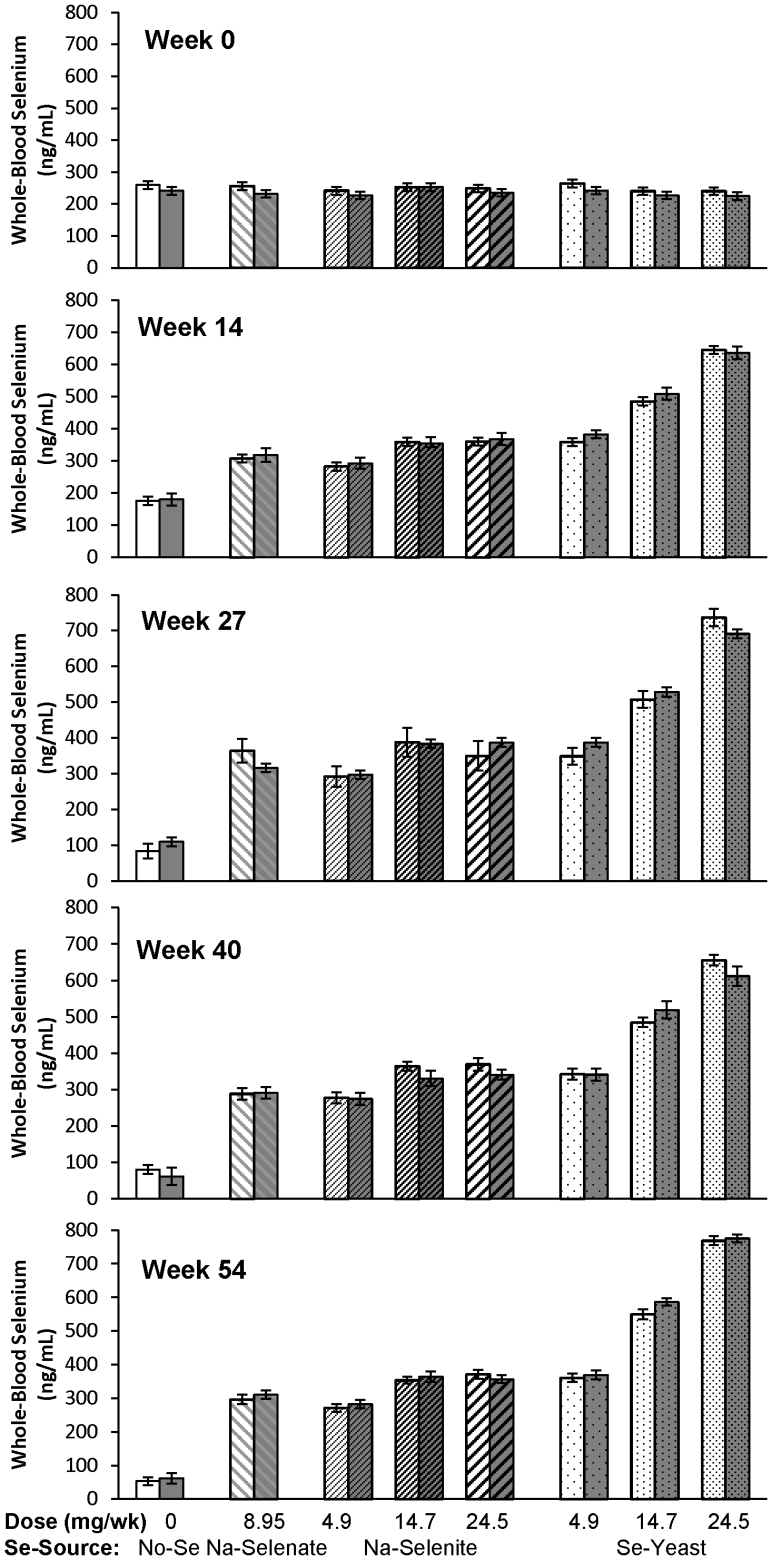
The effect of Se-source, Se-dosage, and foot rot status on whole-blood Se concentrations in sheep. Whole-blood Se concentrations were measured after 0, 14, 27, 40, and 54 wk of Se supplementation, and foot rot severity was assessed after 0, 20, 28, 40, and 60 wk of Se supplementation in ewes receiving no Se treatment, Na-selenate at a dosage rate of 8.95 mg Se/wk per ewe, or Na-selenite and Se-Yeast at 4.9, 14.7, or 24.5 mg Se/wk per ewe for 62 wk. Whole-blood Se concentrations for each treatment group are shown as separate bars for healthy sheep (lighter background) and for sheep with foot rot (darker background). At baseline (wk 0), no significant treatment group differences were observed; however, healthy ewes had higher WB-Se concentrations than ewes with FR (*P* = 0.01). After treatments started, group differences by foot-rot status subsided, whereas Se-source and Se-dosage affected WB-Se concentrations. Both healthy and FR-affected ewes that received no Se treatment had decreased WB-Se concentrations (*P* < 0.0001). Supranutritional Se-yeast supplementation increased WB-Se concentrations linearly with dosage (*P* < 0.0001), whereas ewes receiving supranutritional Na-selenite supplementation at 14.7 and 24.5 mg Se/wk achieved similar WB-Se concentrations as ewes receiving 4.9 mg/wk of Se-yeast.

**Figure 3:**
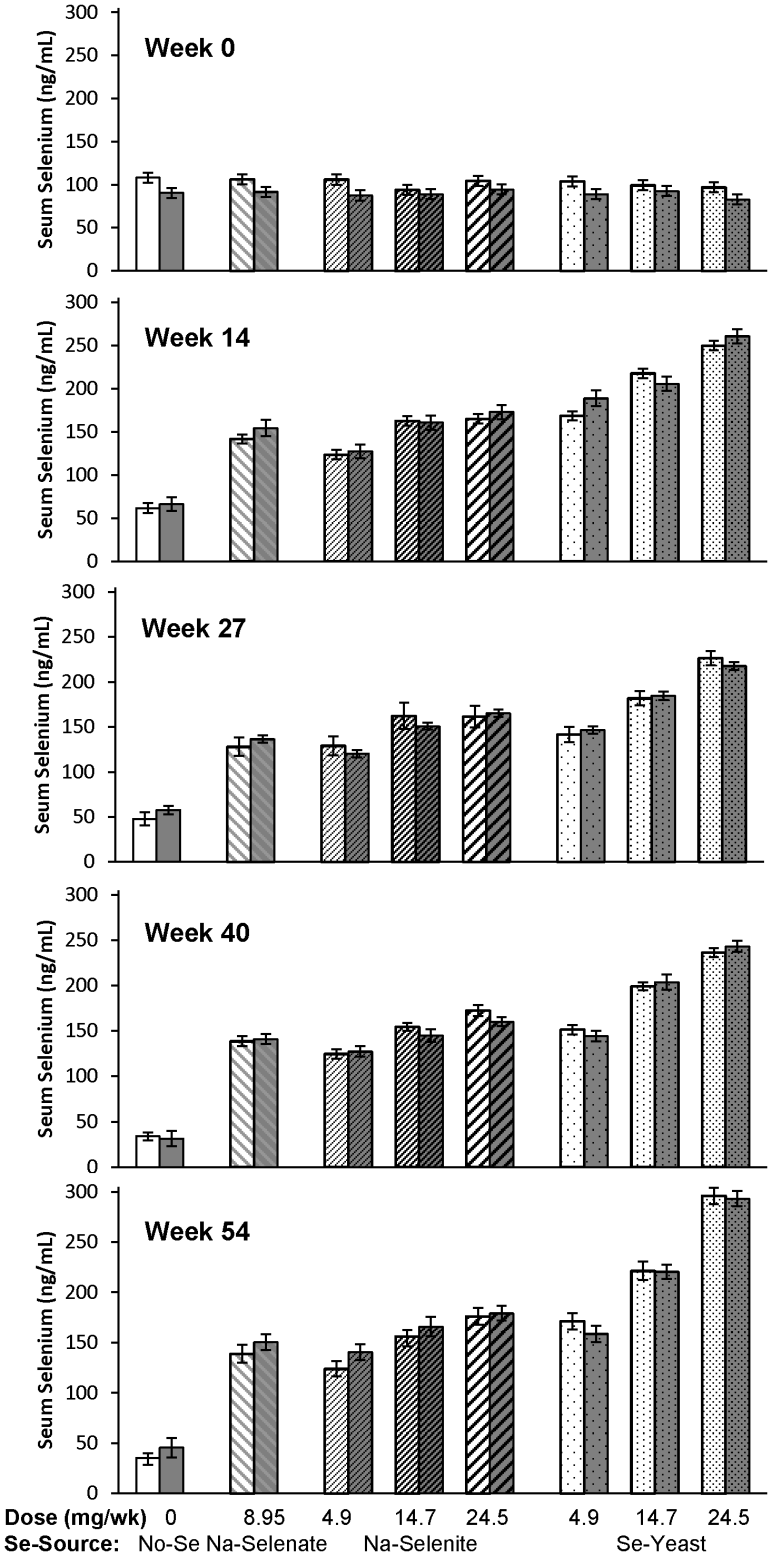
The effect of Se-source, Se-dosage, and foot rot status on serum-Se concentrations in sheep. Serum-Se concentrations were measured after 0, 14, 27, 40, and 54 wk of Se supplementation, and foot rot severity was assessed after 0, 20, 28, 40, and 60 wk of Se supplementation in ewes receiving no Se treatment, Na-selenate at a dosage rate of 8.95 mg Se/wk per ewe, or Na-selenite and Se-Yeast at 4.9, 14.7, or 24.5 mg Se/wk per ewe for 62 wk. Serum-Se concentrations for each treatment group are shown as separate bars for healthy sheep (lighter background) and for sheep with foot rot (darker background). At baseline (wk 0), no significant treatment group differences were observed; however, healthy ewes had higher serum-Se concentrations than ewes with FR (*P* = 0.01). After treatments started, group differences by foot-rot status subsided, whereas Se-source and Se-dosage affected serum-Se concentrations. Both healthy and FR-affected ewes that received no Se treatment had decreased serum-Se concentrations (*P* < 0.0001). Supranutritional Se-yeast supplementation increased serum-Se concentrations linearly with dosage (*P* < 0.0001), whereas ewes receiving supranutritional Na-selenite supplementation at 14.7 and 24.5 mg Se/wk achieved similar serum-Se concentrations as ewes receiving 4.9 mg/wk of Se-yeast.

The effects of Se-source and Se-dosage on WB- and serum Se concentrations are shown in **Table 1** and **Table 2**, respectively. At baseline, healthy ewes had higher WB-Se concentrations than ewes with FR (250 versus 235 ng/mL; *P* = 0.01) and higher serum-Se concentrations (102 versus 90 ng/mL; *P* < 0.0001). After Se supplementation, no significant differences in WB- and serum-Se concentrations were observed between healthy and FR-affected ewes within the same treatment group; this effect was irrespective of Se-source and Se-dosage (**Figure 2** and **Figure** **3**). Both healthy and FR-affected ewes that received no Se treatment had decreased WB-Se and serum-Se concentrations (both *P* < 0.0001). Supranutritional Se-yeast supplementation increased WB-Se and serum-Se concentrations linearly with dosage (*P* < 0.0001), whereas ewes receiving supranutritional Na-selenite supplementation at 14.7 and 24.5 mg Se/wk achieved similar WB-Se concentrations as ewes receiving 4.9 mg/wk of Se-yeast. Ewe FR status did not affect supplementation-induced changes in WB- or serum-Se concentrations.

**Table 1:** Whole-blood Se concentrations (ng/mL) in ewes after weekly oral drenching with no Se, inorganic Se (Na-selenate or Na-selenite), and organic Se (Se-yeast) at varying supplementation rates (4.9, 14.7, and 24.5 mg Se/wk; Na-selenate only at 8.95 mg Se/wk) for 62 wk.^1^

		Oral Selenium Drench								*P*-value
Se-Source	No-Se	Na-Selenate	Na-Selenite			Se-Yeast				
Se-Dosage, mg Se/wk	0	8.95	4.9	14.7	24.5	4.9	14.7	24.5	SEM^2^	Overall
**Week 0**										
Healthy N^3^	15	15	15	15	15	15	15	15		
LSMean^4^	259	258	241	253	249	264	240	240	12	0.75
Footrot N^5^	15	15	15	15	15	15	15	15		
LSMean^4^	241	232	227	253	235	242	227	225	12	0.71
**Week 14**										
Healthy N	19	23	20	21	20	23	19	20		
LSMean	176	307	282	359	360	358	485	645	13	<0.0001
Footrot N	10	7	10	9	9	7	9	9		
LSMean	180	318	293	355	368	383	509	636	22	<0.0001
**Week 27**										
Healthy N	8	4	4	2	3	6	7	7		
LSMean	84	364	292	388	350	349	507	737	41	<0.0001
Footrot N	21	26	26	28	26	23	21	22		
LSMean	109	316	297	361	383	388	529	691	13	<0.0001
**Week 40**										
Healthy N	22	14	16	22	11	15	21	17		
LSMean	50	289	277	364	369	342	485	656	16	<0.0001
Footrot N	6	14	12	8	17	12	6	11		
LSMean	61	292	275	331	341	341	519	612	23	<0.0001
**Week 54**										
Healthy N	18	11	15	21	12	13	10	10		
LSMean	53	296	271	353	371	361	550	768	15	<0.0001
Footrot N	9	13	13	9	15	13	17	14		
LSMean	62	311	282	364	356	369	586	775	15	<0.0001

1 Adapted from [1]; numbers differ because all sheep are included here and classified as healthy or footrot-affected.

2 The largest SEM of the 8 treatment groups is shown.

3 Number of healthy sheep in each treatment group.

4 Least squares means.

5 Number of footrot-affected sheep in each treatment group.

**Table 2:** Serum-Se concentrations (ng/mL) in ewes after weekly oral drenching with no Se, inorganic Se (Na-selenate or Na-selenite), and organic Se (Se-yeast) at varying supplementation rates (4.9, 14.7, and 24.5 mg Se/wk; Na-selenate only at 8.95 mg Se/wk) for 62 wk.^1^

		Oral Selenium Drench										*P*-value
Se-Source	No-Se	Na-Selenate		Na-Selenite				Se-Yeast				
Se-Dosage, mg Se/wk	0	8.95	4.9		14.7	24.5	4.9		14.7	24.5	SEM^2^	Overall
**Week 0**												
Healthy N^3^	15	15	15		15	15	15		15	15		
LSMean^4^	108	106	106		94	104	104		99	97	6	0.62
Footrot N^5^	15	15	15		15	15	15		15	15		
LSMean^4^	90	92	87		89	94	89		92	83	6	0.92
**Week 14**												
Healthy N	19	23	20		21	20	23		19	20		
LSMean	62	142	123		162	165	168		217	250	6	<0.0001
Footrot N	10	7	10		9	9	7		9	9		
LSMean	66	154	127		160	173	189		205	260	9	<0.0001
**Week 27**												
Healthy N	8	4	4		2	3	6		7	7		
LSMean	48	128	129		162	162	142		182	226	15	<0.0001
Footrot N	21	26	26		28	26	23		21	22		
LSMean	58	136	120		151	165	147		185	218	4	<0.0001
**Week 40**												
Healthy N	22	14	16		22	11	15		21	17		
LSMean	34	139	125		154	172	151		199	236	6	<0.0001
Footrot N	6	14	12		8	17	12		6	11		
LSMean	31	141	127		145	160	144		204	243	8	<0.0001
**Week 54**												
Healthy N	18	11	15		21	12	13		10	10		
LSMean	35	139	124		156	176	172		221	296	9	<0.0001
Footrot N	9	13	13		9	15	13		17	14		
LSMean	46	150	141		166	179	159		221	293	10	<0.0001

1 Adapted from [1]; numbers differ because all sheep are included here and classified as healthy or footrot-affected.

2 The largest SEM of the 8 treatment groups is shown.

3 Number of healthy sheep in each treatment group.

4 Least squares means.

5 Number of footrot-affected sheep in each treatment group.

None of the ewes in the no-Se treatment group showed clinical signs of nutritional myodegeneration from Se deficiency [11]. None of the ewes receiving supranutritional Se supplementation showed clinical signs of Se toxicity at any time during the study.

### Foot lesions

**Figure 4:**
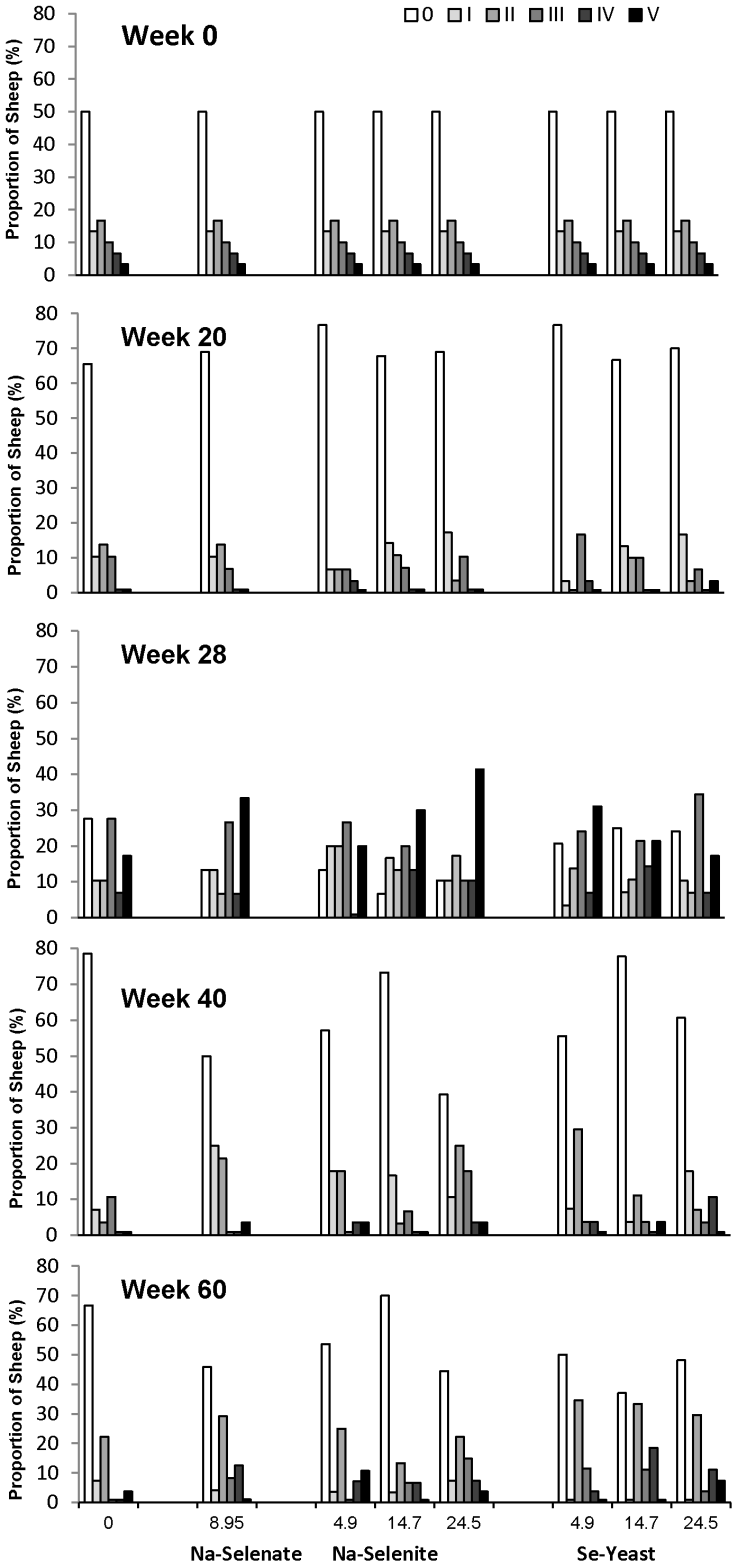
The effect of Se-source and dosage on foot rot (FR) severity in sheep. Foot rot severity was assessed after 0, 20, 28, 40, and 60 wk of Se supplementation in ewes receiving no Se treatment, Na-selenate at a dosage rate of 8.95 mg Se/wk per ewe, or Na-selenite and Se-Yeast at 4.9, 14.7, or 24.5 mg Se/wk per ewe for 62 wk. The proportion of sheep (%) in each FR-severity category within each treatment group is shown (scale 0 to V; with no FR  =  0 being the lowest category). The scoring and categorization for FR is described in detail in the Materials and Methods. There were no consistent effects of Se source and dosage on FR prevalence and severity across time.

**Figure 5:**
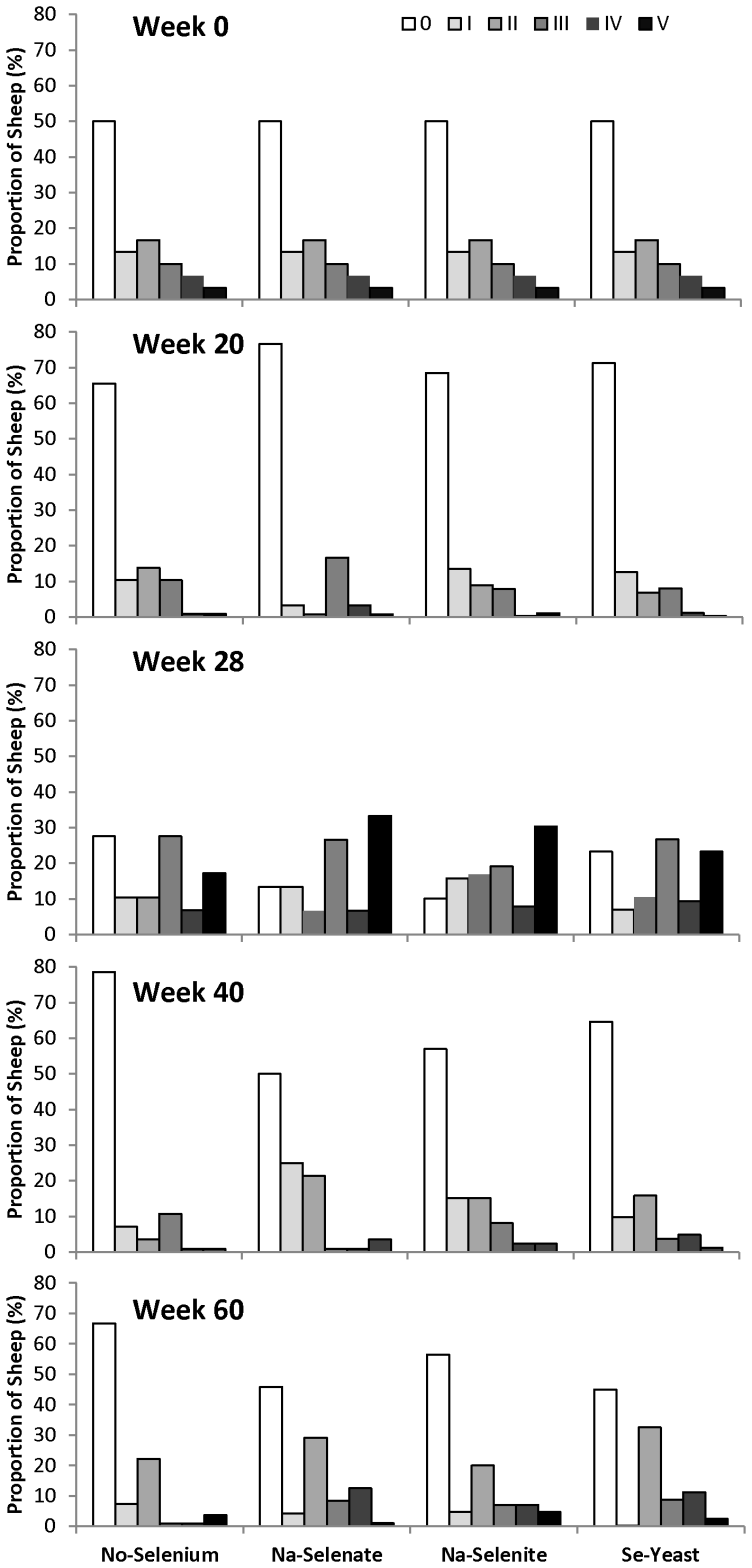
The effect of Se-source on foot rot (FR) severity in sheep. Foot rot severity was assessed after 0, 20, 28, 40, and 60 wk of Se supplementation in ewes receiving no Se treatment, Na-selenate, Na-selenite, and Se-Yeast for 62 wk. All dosage groups were combined for each chemical source of Se treatment. The proportion of sheep (%) in each FR-severity category within each treatment group is shown (scale 0 to V; with no FR  =  0 being the lowest category). The scoring and categorization for FR is described in detail in the Materials and Methods. There were no consistent effects of Se source on FR prevalence and severity across time.

The effects of supplementing ewes with Se on FR severity are shown in **Figure 4** (effects of Se dosage) and **Figure 5** (effects of Se source). The proportion of sheep (%) in each FR-severity category within each treatment group is shown at wk 0, 20, 28, 40, and 60. There were no consistent effects of Se source and dosage on FR prevalence and severity over the 62 week treatment period. Overall, FR severity and prevalence decreased from 0 to 20 wk (50±3% to 30±3%; *P* < 0.0001). The effect of Se treatment on FR prevalence was influenced by the presence of FR disease (*P*_Interaction_ = 0.04). Selenium source and dosage did not influence the interaction. Ewes that were healthy at baseline and received Se treatment had lower FR prevalence at wk 20 (FR score II or greater) than ewes that were healthy at baseline and received no Se (8±3% versus 29±13%, respectively; *P* = 0.03). No differences in FR prevalence were observed in ewes that had FR at baseline and received Se treatment (28±4%) or no Se (20±11%).

After the ewes were moved from pasture into the barn for lambing at wk 20, the FR severity and prevalence increased in all ewes, to 82±2% at wk 28 (*P* < 0.0001). Ewes receiving Se-yeast (77±5%) or no Se (72±8%) had lower FR prevalence than ewes receiving Na-selenite (90±3%; both *P* = 0.02) (**Figure 5**). No dosage-associated differences were observed.

Once the ewes were returned to pasture at wk 30, the FR prevalence decreased from 82±2% at wk 28 to 39±3% at wk 40 (*P* < 0.0001). In ewes previously affected with FR at wk 28, the effect of Se treatment on FR prevalence (FR score II or greater) at wk 40 differed by Se source and dosage. Ewes receiving Na-selenite at increasing dosages responded differently than ewes receiving Se-yeast at increasing dosages (*P*_Interaction_  =  0.02). The FR prevalence was higher in previously FR-affected ewes receiving the highest Na-selenite dosage compared with the two lower Na-selenite dosages (4.9 mg/wk: 21±8%; 14.7 mg/wk: 11±6%; 24.5 mg/wk: 52±10%; *P* = 0.002), but lower with supranutritional Se-yeast supplementation compared with the lowest Se-yeast dosage (4.5 mg/wk: 45±11%; 14.7 mg/wk: 20±9%; 24.5 mg/wk: 32±10%; *P* = 0.04), with values for ewes receiving no Se being 20±9% and for ewes receiving Na-selenate 29±9%.

**Figure 6:**
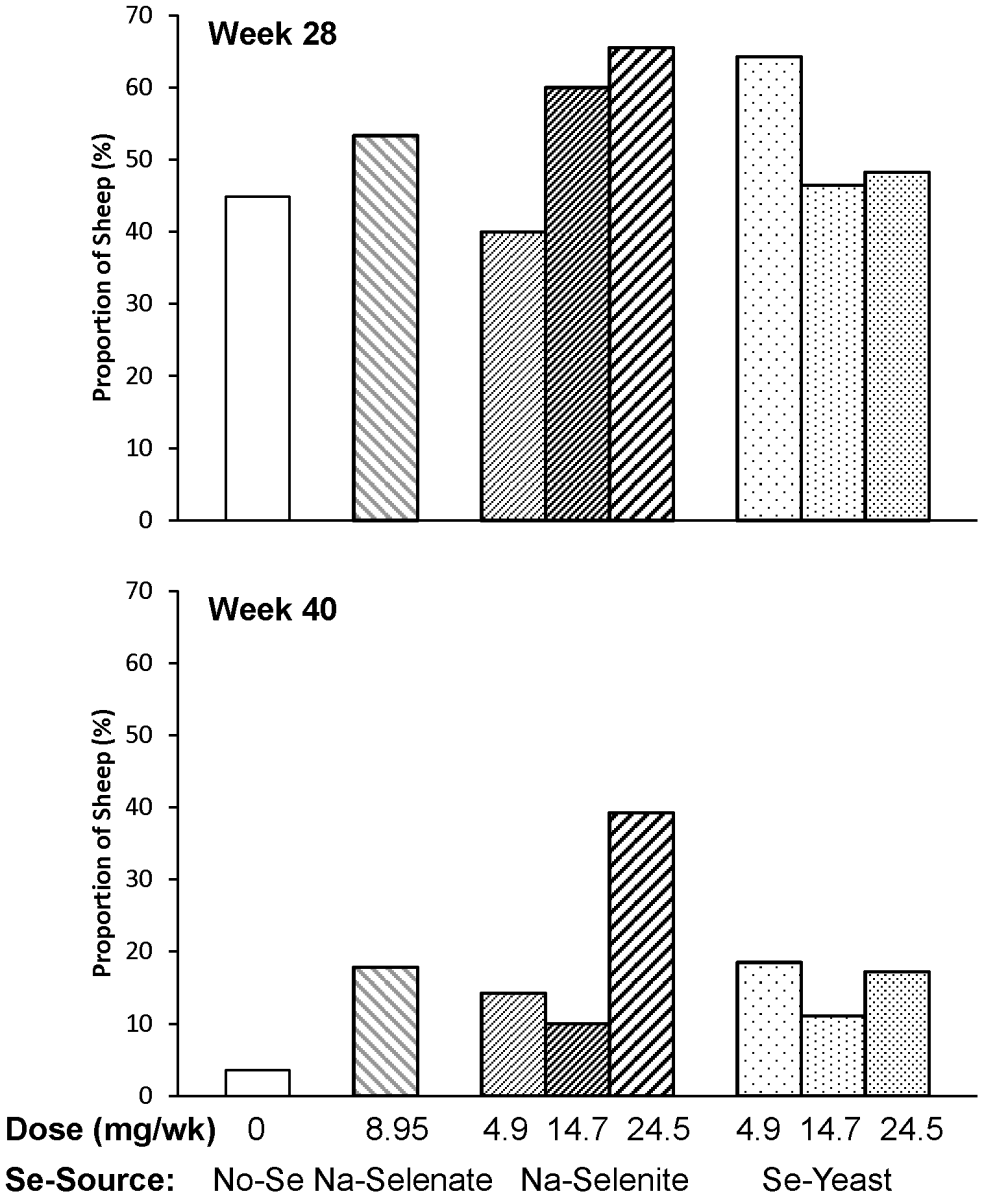
The effect of Se-source and dosage on sheep requiring oxytetracyline treatment. Foot rot severity was assessed after 0, 20, 28, 40, and 60 wk of Se supplementation in ewes receiving no Se treatment, Na-selenate at a dosage rate of 8.95 mg Se/wk per ewe, or Na-selenite and Se-Yeast at 4.9, 14.7, or 24.5 mg Se/wk per ewe for 62 wk. If a sheep had one foot with a score of 4, or one foot with a score of 3 and a second foot with a score of 2 or greater, or if all 4 feet had FR, then 20 mg/kg oxytetracycline was administered subcutaneously (Liquamycin LA-200; Pfizer Animal Health, Exton, PA) at the 28- and 40-wk foot trimming sessions. No significant differences in parental oxytetracycline treatment were observed for individual treatment groups at 28 wk; however; more ewes with supranutritional Na-selenite treatment were treated with oxytetracyline than ewes receiving the lowest Na-selenite dosage (*P* = 0.05). At 40 wk, more ewes receiving Na-selenite at the highest dosage had to be treated with oxytetracycline than ewes receiving no Se, Na-selenite at both lower dosages, or Se-yeast at 14.5 mg/wk (all *P* ≤ 0.05).

At wk 28, the more severely FR-affected ewes were treated with parenteral oxytetracycline (52% of all ewes). This contributed to the overall decrease in FR prevalence at wk 40, to 39±3% (*P* < 0.0001). No significant differences in parental oxytetracycline treatment were observed for individual treatment groups at wk 28 (**Figure 6**); however; more ewes with supranutritional Na-selenite treatment were treated with oxytetracyline than ewes receiving the lowest Na-selenite dosage (*P* = 0.05). In oxytetracycline-treated ewes, the effect of Se treatment on FR prevalence at wk 40 (FR score II or greater) also differed by Se source and dosage. Again, ewes receiving Na-selenite at increasing dosages responded differently than ewes receiving Se-yeast at increasing dosages (*P*_Interaction_ = 0.05), although with fewer numbers of sheep in this oxytetracycline-treatment subgroup, significance was more difficult to demonstrate. In oxytetracyline-treated ewes, FR prevalence at wk 40 tended to be higher in ewes receiving the highest Na-selenite dosage compared with the two lower Na-selenite dosages (4.9 mg/wk: 9±9%; 14.7 mg/wk: 17±9%; 24.5 mg/wk: 39±12%; *P* = 0.06), but lower with supranutritional Se-yeast supplementation compared with the lowest Se-yeast dosage (4.5 mg/wk: 41±12%; 14.7 mg/wk: 31±13%; 24.5 mg/wk: 21±11%; *P* = 0.29). Ewes receiving no Se (33±14%) or Na-selenate (29±13%) had higher FR prevalence than the overall mean of oxytetracycline-treated ewes (28±4%).

The overall FR prevalence increased again between wk 40 and wk 60, to 48±3% (*P* = 0.02), despite a second oxytetracycline treatment of the more severely affected ewes at wk 40 (16% of all ewes), which dropped their FR prevalence (FR score II or greater) from 100% to 59±9%. More ewes receiving Na-selenite at the highest dosage had to be treated with oxytetracycline at wk 40 than ewes receiving no Se, Na-selenite at both lower dosages, or Se-yeast at 14.5 mg/wk (all *P* ≤ 0.05; **Figure 6**). Across all groups, the lowest FR prevalences were observed in ewes receiving no Se (26±9%) and 14.7 mg Na-selenite/wk (27±8%), whereas FR prevalences for the other treatment groups (8.95 mg Na-selenate: 50±8%; 4.5 mg Na-selenite/wk: 43±10%; 24.5 mg Na-selenite/wk: 48±10%; 4.5 mg Se-yeast/wk: 50±10%; 14.7 mg Se-yeast/wk: 63±9%; 24.5 mg Se-yeast/wk: 52±10%) were similar or greater than the overall mean (48±3%). Ewes receiving Se-yeast had on average a greater FR prevalence than ewes receiving Na-selenite (*P* = 0.03) or no Se (*P* = 0.01). Whereas FR prevalence was similar in ewes receiving different dosages of Se-yeast, ewes receiving 14.7 mg Na-selenite/wk had a lower FR prevalence than ewes receiving 4.9 or 24.5 mg Na-selenite (*P* = 0.05).

Overall, the proportion of ewes that had a worse FR score at wk 60 compared with wk 40 did not differ significantly between groups. There was, however, an interaction between Se source and previous FR presence on change in FR score between wk 40 and wk 60 (*P* = 0.02). Whereas in healthy ewes the number with worse scores at wk 60 was lower in ewes receiving no Se and Na-selenate compared with ewes receiving Na-selenite and Se-yeast (no Se: 18±8%; Na-selenate: 14±10%; Na-selenite: 35±7%; Se-yeast: 42±7%; *P* = 0.03), the reverse was true in previously FR affected ewes (no Se: 50±22%; Na-selenate: 50±14%; Na-selenite: 24±7%; Se-yeast: 23±8%; *P* = 0.04). In other words, Na-selenite and Se-yeast treatments decreased FR severity at wk 60 in ewes affected with FR at wk 40 compared with ewes receiving no Se or Na-selenate.

In general, older ewes had a higher FR severity (*P* < 0.0001) and prevalence (*P* < 0.0001). For example, ewes 5 years and older at lambing had a higher FR severity score and FR prevalence than younger ewes at wk 60 (60±5% versus 39±4%, respectively).

### Neutrophil bacterial killing and the 30 minute DTH skin test response are innate immunity measurements

**Figure 7:**
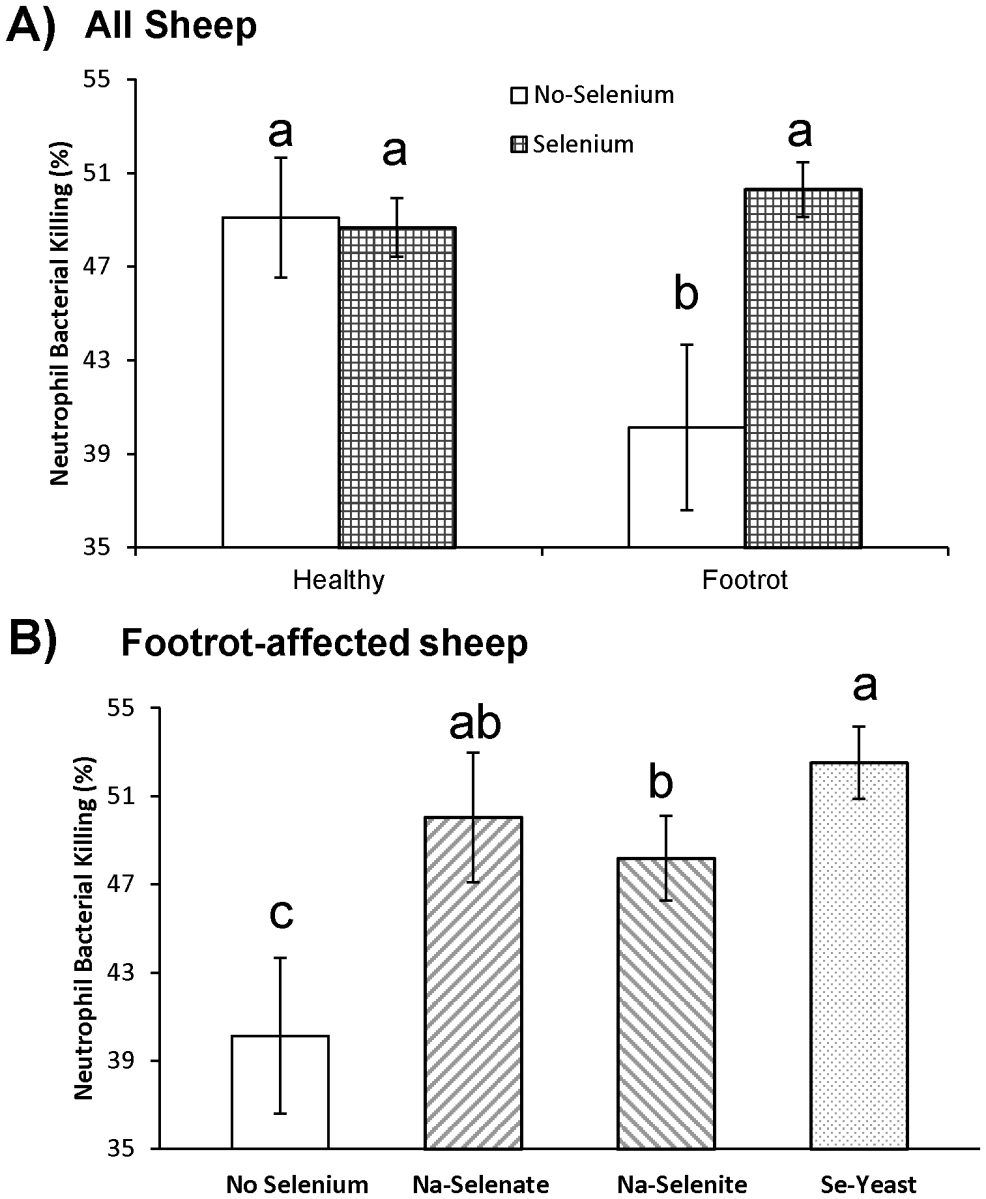
The effect of Se-source on ex vivo neutrophil bacterial killing in sheep. Ex vivo neutrophil bacterial killing was assessed after 60 wk of Se supplementation in healthy and footrot (FR)-affected ewes receiving no Se treatment, Na-selenate, Na-selenite, and Se-Yeast for 62 wk**.** **A**) Weekly oral Se drenching improved neutrophil bacterial killing in FR-affected ewes to percentages that were similar to healthy supplemented or healthy non-supplemented ewes. **B**) In FR-affected ewes, Na-selenate, Na-selenite, or Se-yeast improved neutrophil bacterial killing compared with no Se supplementation. The effect tended to be greater in ewes receiving Se-yeast compared with Na-selenite. The three dosages of Na-selenite or Se-yeast were combined because they did not differ. Different superscripts indicate group differences at *P* ≤ 0.10.

The effect of Se treatment on neutrophil bacterial killing was influenced by the presence of FR disease (*P*_Interaction_ = 0.02; **Figure 7A**). Weekly oral Se drenching improved neutrophil bacterial killing in FR-affected ewes from 40±4% to 50±1% (*P* = 0.007), percentages which were similar to healthy ewes receiving no-Se (49±3%) or healthy ewes receiving Se treatment (49±1%). Compared with no Se treatment, neutrophil bacterial killing was greater in FR-affected ewes receiving Na-selenate, Na-selenite, and Se-yeast (all *P* < 0.05) and tended to be greater in FR-affected ewes receiving Se-yeast compared with Na-selenite (*P* = 0.09; **Figure 7B**). Supranutritional Se treatment did not provide additional benefits, regardless of Se source (data not shown).

Neutrophil bacterial killing decreased linearly in ewes with age (< 5 years: 54±1%; 5 years: 49±2%; >5 years: 43±2%; *P* < 0.0001). The age-associated decline in neutrophil bacterial killing was delayed with Se treatment (< 5 years and no-Se: 50±3%; < 5 years and Se treatment: 54±1%; 5 years and no-Se: 42±5%; 5 years and Se treatment: 50±2%; >5 years and no-Se: 39±4%; >5 years and Se: 44±2%).

**Figure 8:**
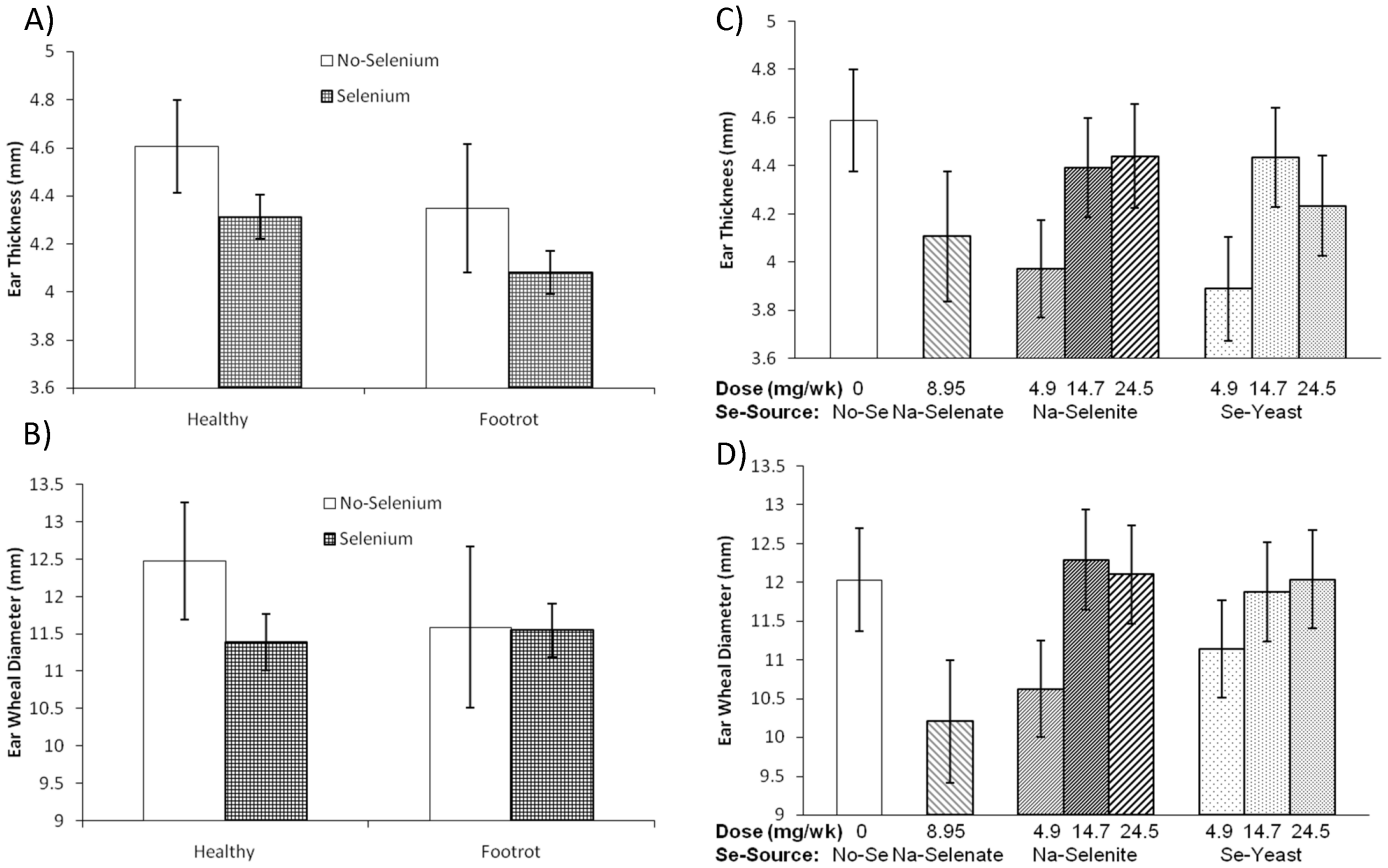
The effect of Se-source and dosage on 30-min skin response to keyhole limpet hemocyanin (KLH). The 30-min skin test response following KLH challenge was assessed after 52 wk of Se supplementation in healthy and footrot (FR)-affected sheep receiving no Se treatment, Na-selenate at a dosage rate of 8.95 mg Se/wk per ewe, or Na-selenite and Se-Yeast at 4.9, 14.7, or 24.5 mg Se/wk per ewe for 62 wk. **A**) Ewes affected with FR had smaller 30 min ear-thickness responses to KLH intradermal injection than healthy ewes (overall *P* = 0.05). **B**) The 30 min ear-wheal diameter response was not significantly affected by ewe FR status or Se supplementation. **C**) Selenium dosage, but not Se source affected the ear-thickness response, as Se supplementation at lower dosages (8.95 mg/wk Na-selenate, 4.9 mg/wk Na-selenite, and 4.9 mg Se-yeast/wk) had smaller ear-thickness responses than supranutritional Se supplementation (14.7 and 24.5 mg/wk Na-selenite and Se-yeast; *P* = 0.01) or no Se supplementation (*P* = 0.01). **D**) Selenium dosage, but not Se source affected the ear-thickness response, as Se supplementation at lower dosages had or tended to have smaller ear-wheel diameter responses than supranutritional Se supplementation (*P* = 0.03) or no Se supplementation (*P* = 0.07).

The effect of FR status on 30 minute responses to KLH was not consistent. Ewes affected with FR had smaller 30 min ear-thickness responses to KLH intradermal injection than healthy ewes (4.11±0.08 versus 4.35±0.09 mm; *P* = 0.05), which was not influenced by Se treatment (*P*_Interaction_ = 0.94; **Figure 8A**). Such an effect was not observed for 30 min ear-wheal diameter responses to KLH injection (*P* = 0.95; **Figure 8B**). Selenium dosage, but not Se source affected 30 minute responses to KLH. Se supplementation at lower dosages (8.95 mg/wk Na-selenate, 4.9 mg/wk Na-selenite, and 4.9 mg Se-yeast/wk) had smaller ear-thickness responses than supranutritional Se supplementation (14.7 and 24.5 mg/wk Na-selenite and Se-yeast; *P* = 0.01) or no Se supplementation (*P* = 0.01; **Figure 8C**). A similar effect was observed for ear-wheal diameter response, as Se supplementation at lower dosages had or tended to have smaller ear-wheel diameter responses than supranutritional Se supplementation (*P* = 0.03) or no Se supplementation (*P* = 0.07; **Figure 8D**). In addition, Polypay ewes had smaller ear-wheal diameter responses than Suffolk and Suffolk cross ewes (10.1±0.3 versus 13.0±0.4 mm; *P* < 0.0001).

### Cell-mediated immunity as measured by the delayed-type hypersensitivity (DTH) skin test with KLH

**Figure 9:**
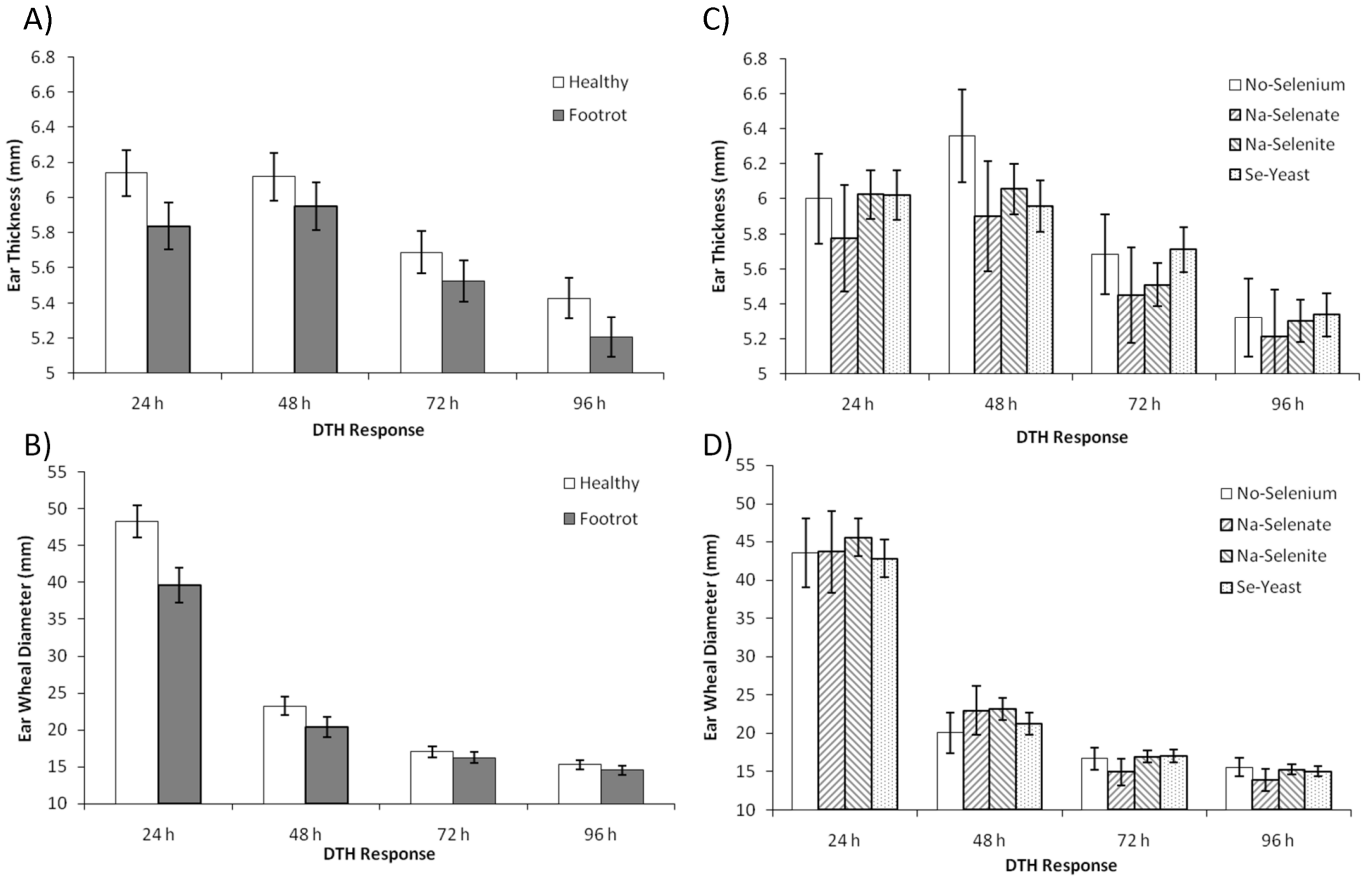
The effect of Se-source and dosage on delayed-type hypersensitivity response to keyhole limpet hemocyanin (KLH). Delayed-type hypersensitivity (DTH) skin test responses was assessed after 52 wk of Se supplementation in healthy and footrot (FR)- affected sheep receiving no Se treatment, Na-selenate at a dosage rate of 8.95 mg Se/wk per ewe, or Na-selenite and Se-Yeast at 4.9, 14.7, or 24.5 mg Se/wk per ewe for 62 wk. (All Se treatment groups were combined.) **A**) Ear thickness response was not significantly affected by ewe FR status (*P* = 0.15). **B**) Ear wheal diameter response was decreased in FR-affected sheep (overall *P* = 0.03), which was significant only at 24 h (*P* = 0.007; *P*_Interaction_ = 0.05). C) Ear thickness response and D) ear wheal diameter response were not significantly affected by Se source or Se dosage.

FR-affected sheep demonstrated suppressed CMI at 24 h after intradermal KLH challenge. For the DTH ear thickness response, there was no significant effect of ewe FR status (*P* = 0.15; **Figure 9A**). Significance was achieved, however, for the DTH ear wheal diameter response as sheep affected with FR had an attenuated DTH response compared with healthy sheep (overall *P* = 0.03), which was significant only at 24 h (39.6±2.3 versus 48.2±2.2 mm, respectively; *P* = 0.007; *P*_Interaction_ = 0.05; **Figure 9B**). Selenium source and dosage did not significantly alter the ear thickness response (**Figure 9C**) or the ear wheal diameter response (**Figure 9D**).

### Humoral immunity as measured by the KLH antibody titer

**Figure 10:**
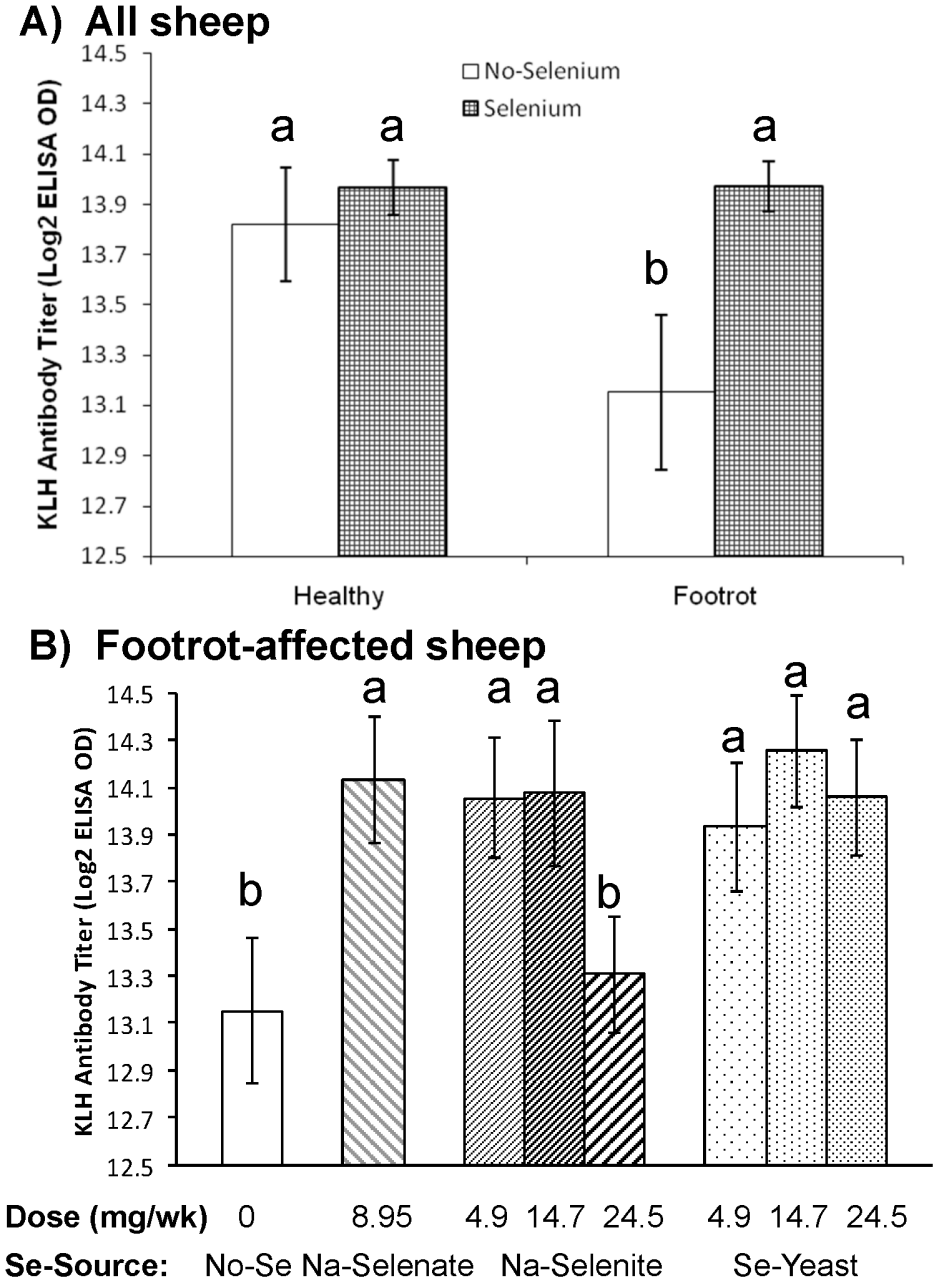
The effect of Se-source and dosage on antibody titers to keyhole limpet hemocyanin (KLH). Antibody titers to keyhole limpet hemocyanin (KLH) were assessed after 60 wk of Se supplementation in healthy and footrot (FR)-affected sheep receiving no Se treatment, Na-selenate at a dosage rate of 8.95 mg Se/wk per ewe, or Na-selenite and Se-Yeast at 4.9, 14.7, or 24.5 mg Se/wk per ewe for 62 wk. A) Weekly oral Se drenching improved KLH antibody titers in FR-affected ewes to titers that were similar to healthy ewes receiving no Se or healthy ewes receiving Se treatment. **B**) In FR-affected ewes, KLH antibody titers were greater in all Se-supplemented groups compared with the non supplemented group with the exception of the 24.5 mg/wk Na-selenite group. Different superscripts indicate group differences at *P* ≤ 0.10.

Similar to the results for neutrophil bacterial killing, the effect of Se treatment on KLH antibody titer tended to be influenced by the presence of FR disease (*P*_Interaction_ = 0.09; **Figure 10A**). Weekly oral Se drenching improved KLH antibody titer in FR-affected ewes from 13.15±0.31 to 13.97±0.10 (*P* = 0.01), titers which were similar to healthy ewes receiving no-Se (13.82±0.22) or healthy ewes receiving Se treatment (13.97±0.11). Compared with no Se treatment, KLH antibody titers were greater in all Se-treatment groups (all *P* < 0.06) except for the 24.5 mg/wk Na-selenite group (**Figure 10B**).

## Discussion

### Effect of dietary Se depletion, different dietary Se sources, and supranutritional Se-supplementation dosages on immune responses in healthy and FR-affected ewes

The immune system has two functional divisions: innate and adaptive immunity. Both divisions involve various blood-borne factors (e.g., complement, antibodies, and cytokines) and cells (e.g., neutrophils, lymphocytes, and macrophages). Neutrophils are the most numerous and important cellular component of innate immunity. Their primary functions are phagocytosis and destruction of microorganisms. They serve as the body’s first line of defenses against invading microorganisms. Phagocytosed bacteria are rapidly killed by proteolytic enzymes (e.g., myeloperoxidase), antimicrobial proteins, and ROS when membrane-bound granules fuse with phagocytic vesicles. To assess innate immunity of neutrophils, an *ex vivo* biologic assay was performed using *E. coli* and measuring percent bacterial killing. Neutrophils from healthy sheep not receiving Se supplementation demonstrated higher percent bacterial killing compared with neutrophils from FR-affected sheep not receiving Se supplementation, consistent with our results published previously for another flock [26]. In the current study we were able to show that Se supplementation, regardless of source or dosage, restored neutrophil bacterial killing in FR-affected ewes back to percentages consistent with Se-supplemented or non-supplemented healthy ewes. The bacterial killing percentage for neutrophils tended to be greater in FR-affected ewes receiving Se-yeast compared with Na-selenite. We saw no clear benefit from supranutritional Se dosages in neutrophil bacterial killing.

In a companion paper [31], we reported on neutrophil-related gene expression profiles from these ewes and showed a U-shaped relationship with supranutritional Se-yeast supplementation and Se depletion both enhancing gene expression of L-Selectin (L-Sel), interleukin 8 receptor (IL-8R), and toll-like receptor 4 (TLR4). All three are essential for bacterial recognition and neutrophil migration, phagocytosis, and killing. In addition, expression of selenoprotein S (SEPS1) and glutathione peroxidase 4 (GPx4), which are both involved in controlling inflammation, was increased for both with supranutritional Se-yeast supplementation and Se depletion [31]. When we correlated (nonparametric spearman correlation) neutrophil bacterial killing activity in this study with the previously reported neutrophil gene expression profiles for ewes receiving 0, 4.9, 14.7, and 24.5 mg Se/wk, significant negative correlations were observed for GPx4 (r = −0.24; *P* = 0.01) and SEPS1 (r = −0.26; *P* = 0.01). Both genes act as anti-inflammatory agents: GPx4 promotes cell survival and blocks eicosanoid synthesis, including cyclooxygenase (COX) II [36], [37], and SEPS1 protects immune cells from apoptosis and decreases the release of the proinflammatory cytokines IL-6 and TNF-α [38], [39]. Others have shown in mice that moderate selenium deficiency down-regulates inflammation-related genes and reduces myeloperoxidase and lysozyme activities in Se-restricted leukocytes [40]. Myeloperoxidase is expressed in neutrophils and monocytes and generates ROS that are important for antimicrobial and cytotoxic effects. Thus, Se-supplementation may restore optimal neutrophil bacteria killing in the presence of FR disease, but healthy ewes may have different requirements for dietary Se.

Neutrophil bacterial killing also decreased linearly in ewes with age. Ewes six years and older (43%) had similar killing percentages as FR-affected ewes receiving no Se supplement (40%). Se supplementation delayed the age-associated decline in neutrophil killing ability, as ewes receiving Se had similar killing percentages as non supplemented ewes that were one age category younger. We have previously shown in aged Beagle dogs that older dogs have a significant decrease in neutrophil bacterial killing and, in addition, have lower levels of mRNA for neutrophil-related gene expression compared with younger dogs, including mRNA for myeloperoxidase [41], which may contribute to increased morbidity and mortality with aging. These results are consistent with our finding in this study that older ewes had a higher FR severity and prevalence. Neutrophils from older humans have also been shown to have less phagocytic ability than those from younger adults, and the respiratory burst was altered in neutrophils from aged participants [42].

Tests used to assess adaptive immunity include measuring an antibody titer in response to sensitization/immunization (humoral immune response). The ewe is injected with a novel protein (e.g., KLH) that elicits an immune response. Following sensitization, antibody titers to KLH are measured. Consistent with our results in another flock [26] and results for bacterial killing by neutrophils in the current study, healthy ewes receiving no Se supplementation had higher KLH antibody titers compared with FR affected ewes receiving no Se supplementation. In the current study, we were also able to show that Se supplementation, regardless of source, restored KLH antibody titers in FR-affected ewes back to titers consistent with Se-supplemented and non-supplemented healthy ewes. Others have shown that Se supplementation increases antibody production in Se deficient sheep (reviewed in [16]). Our results suggest that Se supplementation may also improve antibody titers in response to a novel antigen in Se-replete yet FR-affected sheep, which is consistent with results we demonstrated in Se-replete adult beef cattle [16] and weaned beef calves (paper under review).

We did see a disadvantage of supranutritional Se treatment with Na-selenite (24.5 mg/wk dosage) in that KLH titers in FR-affected ewes receiving this Se source and dosage remained suppressed. We also observed at this Na-selenite dosage a higher propensity for FR lesions at several time points. Furthermore, WB-Se concentrations did not increase from the 14.7 mg/wk to the 24.5 mg/wk Na-selenite. In a companion paper, we reported a lower transfer of IgG from ewe colostrum to lamb serum if ewes received 24.5 versus 4.9 mg Se/wk as Na-selenite. [18]. These results suggest that Na-selenite may have potentially deleterious effects at higher dosages. In support, comparative toxicosis studies in sheep showed that oxidative effects were greater for Na-selenite than equivalent amounts of SeMet [43], which is the main selenocompound in Se-yeast [44]. Thus, the efficacy of supranutritional treatment with Na-selenite at 5 times the maximal FDA-permitted level requires further study.

The DTH test, which is also known as a type IV hypersensitivity reaction, is another test used to assess the adaptive immune response. This test provides a general measure of CMI. Professional antigen presenting cells, e.g., dendritic cells, present antigen to T lymphocytes. This results in antigen specific activation of T lymphocytes in local tissues. Inflammatory cytokines produced by these stimulated T lymphocytes cause other mononuclear cells (lymphocytes and macrophages) to migrate to the area and proliferate. To perform this test, foreign antigen is injected under the epidermis of the skin. The immune system responds to this antigen by producing a small raised wheal that can be measured 24 to 96 h after injection. The larger and thicker the wheal, the greater is the DTH response.

In our study, FR-affected sheep demonstrated suppressed CMI at 24 h after intradermal KLH challenge, consistent with our results in another flock [26]. In the current study, this response was significant using ear wheal diameter measurements, and although numerically true for ear thickness measurements, the latter results were not significant. We reported previously in another flock that FR-affected ewes with WB-Se concentrations above 250 ng/mL at the time of the DTH assay had greater ear thickness and ear wheal diameter responses than FR-affected ewes with WB-Se concentrations below 250 ng/mL [26]. In the current study we saw no effect of Se source and dosage on DTH responses. The DTH test may be too insensitive under field conditions to detect a difference in CMI with Se supplementation. It is well known that large variation exists in immune function measures, even among healthy animals. For example, differences in genetics, age, diet, body condition scores, stress, levels of exercise, and infectious disease history are important contributors to observed variation [8], such that demonstrating a consistent improvement in immune function with Se supplementation is challenging. In addition, differences in DTH methodology, with variable injection sites, response times, and measurement techniques may account for differences between studies. Nonetheless, finding once again an attenuated T-lymphocyte response in FR-affected sheep is important, and could be the result of decreased activation, migration, proliferation, or a combination of these effects. Se supplementation alone may or may not (current study) be sufficient to improve the DTH response under FR-disease conditions.

We also assessed the 30-min skin test response following intradermal KLH challenge in healthy and FR-affected sheep to determine if results differed for the type I hypersensitivity reaction normally induced by histamine and inflammatory cytokines. The KLH antigen stimulates inflammatory cytokine production. We found that FR-affected ewes had attenuated 30 min ear-thickness responses to KLH intradermal injection compared with healthy ewes, consistent with results of a previous study [26]. The KLH response was not influenced by source of Se treatment. Footrot bacterial infection could suppress the Type I hypersensitivity response by affecting the release of histamine, or virulence factors such as leukotoxin, endotoxin, haemolysin, haemagglutinin, and adhesion.

Histamine normally increases capillary permeability and relaxes vascular smooth muscle, allowing edema fluid accumulation. Influx of proinflammatory cytokines triggers production of ROS. When produced in excess, ROS are important mediators of cell and tissue injury (reviewed in Murr et al. [20]). As a component of the glutathione peroxidase family of enzymes, Se contributes to the reduction of hydroperoxides in cells. Glutathione peroxidase reduces ROS to less reactive metabolites, decreasing oxidant stress. Because Se is involved in redox reactions, and immune activation is usually associated with increased production of ROS by cells of the immune system, we hypothesized that ewes receiving supranutritional Se supplementation might have suppressed 30 min skin reactions compared with ewes receiving less Se supplementation. . We observed, however, a U-shaped relationship between Se dosage and the 30-min skin test responses to KLH, similar to what we reported in a companion paper for Se-yeast dosage and neutrophil gene expression [31]. Ewes receiving either no Se supplementation or supranutitional Se supplementation both had accentuated rather than suppressed 30-min skin test responses to KLH compared with ewes receiving the maximum FDA-allowed levels. A U-shaped relationship between Se status and human health has been postulated in a review by Rayman [13], whereby supplemental Se intake may benefit people with low Se status (or in our case under certain disease conditions such as FR), but cautions that those with adequate to high Se status might be affected adversely and should not take Se supplements. Decreased inflammation and inflammation-dependent plasma cell tumors have been reported in Se-deficient mice [45]. Both Se deficiency and high levels of Se have been reported to decrease the incidence and progression of liver tumors in transgenic mice prone to liver cancer [46], [47]. A vigorous 30-min skin response to a novel antigen, induced by histamine and proinflammatory gene products, may be necessary for a successful defense against FR-causing bacteria, as FR-affected sheep had a lower 30-min skin test response than healthy sheep in this and another flock [26]. Our results suggest that optimal 30 min skin test responses vary depending on the underlying disease condition and the Se supplementation rate.

### Effect of dietary Se sources and supranutritional Se-supplementation on whole-blood and serum-Se concentrations and FR morbidity in healthy and FR-affected ewes

In the current study, ewes affected with FR at baseline had lower WB- and serum-Se concentrations compared with healthy ewes, although mean concentrations were within the normal reference interval for adult sheep. [The normal reference interval for Se in WB of adult sheep > 700 days of age at the Michigan State University diagnostic laboratory is 150 to 500 ng/mL (T. Herdt, personal communication)]. This finding is consistent with our previously published observations in another flock [48]. In a companion paper, we reported that WB-Se and serum-Se concentrations increased linearly with supranutritional Se-yeast supplementation [1]. However, WB-Se concentrations in ewes receiving supranutritional Na-selenite supplementation reached a plateau similar to concentrations attained in ewes receiving 4.9 mg/wk of Se-yeast [1]. In the current analysis, we showed that ewe FR status does not affect Se-supplementation induced increases in WB- or serum-Se concentrations irrespective of Se-source and Se-dosage. Thus, it is more likely that Se intake was lower in ewes with FR compared with healthy ewes at baseline because sheep affected with FR are less mobile and, therefore, unable or unwilling to consume as much Se-containing mineral supplement as healthy sheep [31] than the alternative hypothesis that Se requirements are higher in the presence of an infectious disease like FR because more Se is required for removal of reactive oxygen species (ROS) associated with inflammation [49].

We previously reported in another sheep flock that parenteral Se-supplementation in conjunction with routine control practices accelerated recovery from FR in sheep [48]. In the current study, even though we were able to raise WB- and serum-Se concentrations, Se supplementation did not consistently prevent FR nor accelerate recovery from FR over the 62 week treatment period compared with no Se supplementation. Selenium supplementation did decrease the percentage of healthy ewes that acquired FR during the first 20 weeks of the study; however, we did not observe a similar effect in wks 28, 40, and 60. In a companion paper, we reported that the greatest treatment success with oxytetracycline was observed in ewes receiving the highest Se-yeast dosage (24.5 mg Se/wk) compared with Se-yeast dosages of 0, 4.9, or 14.5 mg Se/wk [31]. In the current analysis, we observed an improvement in FR prevalence in FR affected sheep receiving Na-selenite and Se-yeast supplementation compared with no Se and Na-selenate supplementation at wk 60, but not at earlier time points.

In general, the prevalence of FR changed according to the ewe production cycle and management practices. At baseline, ewes were selected for 50% FR prevalence, and treatment groups were stratified for FR severity and age of ewe. Ewes were kept on pastures and supplemented with grass hay during the breeding and gestation seasons. Footrot prevalence decreased to 30±2% during this period. Ewes were moved from pasture into the barn for lambing around wk 20 and fed alfalfa hay and shelled corn. By wk 28 the FR prevalence had increased to 82±2%. Serum Se concentrations were decreasing by week 27 after increasing from week 0 to week 14. The decrease in blood Se concentrations reflected an increased Se transfer from ewes to lambs in late gestation and early lactation [1]. A corresponding decrease in WB-Se concentrations was not observed until week 40, likely because of the longer half-life of RBC.

Housing ewes in the barn at higher stocking density for the 3-month period around lambing (up to week 30) likely contributed to increased FR prevalence and severity. It is known that environmental factors play an important role in determining infection rate and progression of FR severity, with heavier infection rates occurring under warm moist conditions [50]-[52]. *Dichelobacter nodosus*, one of the main organisms associated with FR is an anaerobic and fastidious bacterium that colonizes the interdigital epithelial tissue more readily during the wet seasons of spring, fall, and winter. Higher stocking density is also more conducive to heavy infection rates [51]. Crowding and moist bedding conditions could explain the higher FR prevalence in the flock in general during this time period.

Once the ewes were returned to pasture and eating grass forage at wk 30, the FR prevalence decreased to 39±3% at wk 40. In part, this reflects the administration of oxytetracycline antibiotic to the more severely FR-affected ewes at wk 28 (52% of all ewes received oxytetracycline). Again, at wk 40, oxytetracycline treatment of the more severely affected ewes (16% of all ewes) was repeated. Although beneficial in helping decrease FR prevalence in these ewes from 100% to 59±9%, the overall FR prevalence increased between wk 40 and wk 60, to 48±3%.

Selenium is not recommended as the sole treatment for FR in sheep. Administration of topical or systemic antibiotics, foot paring, foot bathing in disinfectants, and vaccination with a commercially available vaccine for footrot (Footvax, MSD) containing multiple serotypes of *D. nodosus*, reviewed by Duncan et al. [53], have all been suggested for use in treating sheep with advanced FR lesions. Ewes that do not respond to treatments are often culled; this was not the case in our study, which explains why older ewes represented the majority of cases of FR infection in our study and another [54]. Early detection of disease and prompt treatment with parenteral long-term acting oxytetracycline were control measures resulting in sheep being significantly more likely to recover from FR lesions and lameness within 5 days of treatment compared with sheep that were foot trimmed with or without parenteral administration of antibacterials [55]. In our study, ewes with FR severity scores of II (with foot scores of 4) and higher received parenteral long-term acting oxytetracycline injections at 28 and 40 wk. In hindsight, a more intensive parenteral antibiotic treatment regimen in the current study may have been beneficial in decreasing pathogen load and, thus, infection challenge. This might have allowed us the opportunity to see more benefits associated with Se supplementation.

It is unclear why ewes receiving no Se supplementation were so resistant to FR infection. One explanation is that dietary Se status was not the most limiting factor for FR infection in this flock. Footrot is a multifactorial disease [51] and, therefore, the optimal Se supplementation dosage may vary depending on nutritional and management conditions, as well as the sheep’s immune system. Another explanation is that inflammation, and thus progression of foot lesions with marginal Se deficiency, is dampened. In support, we have shown in a companion paper [31] that WB-neutrophil gene expression profiles are shifted in an anti-inflammatory direction (increased GPx4 and SEPS1) with no Se supplementation. Others have shown that moderate Se deficiency in mice down-regulates inflammation-related genes and reduces myeloperoxidase and lysozyme activities in Se-restricted leukocytes [40]. Myeloperoxidase is expressed in neutrophils and monocytes and generates ROS that are important for antimicrobial and cytotoxic effects, as well as modulation of the immune response via nuclear factor kappa B (NF-κB) signaling. Down regulation of inflammation under marginal Se deficiency requires further study.

The goal of immunonutrition is to enhance immunity and increase resistance to disease. We are interested in supranutritional levels of Se, to determine if supplementing Se at concentrations above those currently recommended for sheep (supranutritional) can modulate the immune response in a way that reduces the severity and/or improves recovery from a disease process. Using FR as our disease model, we have shown in a companion paper that supranutritional supplementation of these ewes with Se-yeast at 24.5 mg Se/wk improved lamb growth and ewe health compared with maximal FDA-allowed levels of Se-yeast [17]. In this study, Se supplementation did not prevent FR, but did improve innate and humoral immune functions negatively affected by FR. Future studies are warranted to evaluate whether Se supplementation enhances innate and adaptive immune responses and provides protection against other bacterial or viral pathogens.

## References

[1] Hall JA, Van Saun RJ, Bobe G, Stewart WC, Vorachek WR, et al (2012) Organic and inorganic selenium: I. Oral bioavailability in ewes. J Anim Sci 90: 568–576. 21965451 10.2527/jas.2011-4075

[2] Qin SY, Gao JZ, Huang KH (2007) Effects of different selenium sources on tissue selenium concentrations, blood GSH-Px activities and plasma interleukin levels in finishing lambs. Biological Trace Element Research 116: 91–102. 17634631 10.1007/BF02685922

[3] Davis PA, McDowell LR, Wilkinson NS, Buergelt CD, Van Alstyne R, et al (2006) Tolerance of inorganic selenium by range-type ewes during gestation and lactation. Journal of animal science 84: 660–668. 16478958 10.2527/2006.843660x

[4] Steen A, Strom T, Bernhoft A (2008) Organic selenium supplementation increased selenium concentrations in ewe and newborn lamb blood and in slaughter lamb meat compared to inorganic selenium supplementation. Acta Veterinaria Scandinavica 50. 10.1186/1751-0147-50-7 PMC2346462 18377659

[5] Taylor JB, Reynolds LP, Redmer DA, Caton JS (2009) Maternal and fetal tissue selenium loads in nulliparous ewes fed supranutritional and excessive selenium during mid- to late pregnancy. Journal of Animal Science 87: 1828–1834. 19151151 10.2527/jas.2008-1534

[6] Rooke JA, Robinson JJ, Arthur JR (2004) Effects of vitamin E and selenium on the performance and immune status of ewes and lambs. Journal of Agricultural Science 142: 253–262.

[7] Kiremidjian-Schumacher L, Stotzky G (1987) Selenium and immune responses. Environ Res 42: 277–303. 3552651 10.1016/s0013-9351(87)80194-9

[8] Finch JM, Turner RJ (1996) Effects of selenium and vitamin E on the immune responses of domestic animals. Res Vet Sci 60: 97–106. 8685547 10.1016/s0034-5288(96)90001-6

[9] McClure SJ (2008) How minerals may influence the development and expression of immunity to endoparasites in livestock. Parasite Immunol 30: 89–100. 18186769 10.1111/j.1365-3024.2007.00996.x

[10] Hefnawy AE, Tortora-Perez JL (2010) The importance of selenium and the effects of its deficiency in animal health. Small Ruminant Research 89: 185–192.

[11] Muth OH, Oldfield JE, Remmert LF, Schubert JR (1958) Effects of selenium and vitamin E on white muscle disease. Science 128: 1090. 10.1126/science.128.3331.1090 13592294

[12] FDA (2012). Code of Federal Regulations Title 21 - Food and Drugs Chapter 1 -Food and Drug Administration, Department of Health and Human Services Subchapter E - Animal drugs, feeds, and related products Part 573 - Food additive permitted in feed and drinking water of animals Subpart B - Food Additive Listing Section 573920 - Selenium

[13] Rayman MP (2012) Selenium and human health. Lancet 379: 1256–1268. 22381456 10.1016/S0140-6736(11)61452-9

[14] Fairweather-Tait SJ, Collings R, Hurst R (2010) Selenium bioavailability: current knowledge and future research requirements. Am J Clin Nutr 91: 1484S–1491S. 20200264 10.3945/ajcn.2010.28674J

[15] Zeng H, Combs GF Jr (2008) Selenium as an anticancer nutrient: roles in cell proliferation and tumor cell invasion. J Nutr Biochem 19: 1–7. 17588734 10.1016/j.jnutbio.2007.02.005

[16] Hall JA, Harwell AM, Van Saun RJ, Vorachek WR, Stewart WC, et al (2011) Agronomic biofortification with selenium: Effects on whole blood selenium and humoral immunity in beef cattle. Animal Feed Science and Technology 164: 184–190.

[17] Stewart WC, Bobe G, Pirelli GJ, Mosher WD, Hall JA (2012) Organic and inorganic selenium: III. Ewe and progeny performance. J Anim Sci 90: 4536–4543. 22767089 10.2527/jas.2011-5019

[18] Stewart WC, Bobe G, Vorachek WR, Stang BV, Pirelli GJ, et al (2013) Organic and inorganic selenium: IV. Passive transfer of immunoglobulin from ewe to lamb. J Anim Sci 91: 1791–1800. 23408818 10.2527/jas.2012-5377

[19] Egerton JR, Roberts DS, Parsonson IM (1969) The aetiology and pathogenesis of ovine foot-rot. I. A histological study of the bacterial invasion. Journal of comparative pathology 79: 207–215. 5813556 10.1016/0021-9975(69)90007-3

[20] Roberts DS, Egerton JR (1969) The aetiology and pathogenesis of ovine foot-rot. II. The pathogenic association of Fusiformis nodosus and F. necrophorus. Journal of comparative pathology 79: 217–227. 5814119 10.1016/0021-9975(69)90008-5

[21] Bennett G, Hickford J, Sedcole R, Zhou H (2009) Dichelobacter nodosus, Fusobacterium necrophorum and the epidemiology of footrot. Anaerobe 15: 173–176. 19239925 10.1016/j.anaerobe.2009.02.002

[22] Elleman TC, Hoyne PA, Emery DL, Stewart DJ, Clark BL (1984) Isolation of the gene encoding pilin of Bacteroides nodosus (strain 198), the causal organism of ovine footrot. FEBS letters 173: 103–107. 6146535 10.1016/0014-5793(84)81026-1

[23] Green LE, George TR (2008) Assessment of current knowledge of footrot in sheep with particular reference to Dichelobacter nodosus and implications for elimination or control strategies for sheep in Great Britain. Veterinary journal 175: 173–180. 10.1016/j.tvjl.2007.01.014 17418598

[24] Mitchell R (2003) Footrot Eradication in Western Australia. Department of Agriculture Western Australia, Scott Print.

[25] Kaler J, Daniels SL, Wright JL, Green LE (2010) Randomized clinical trial of long-acting oxytetracycline, foot trimming, and flunixine meglumine on time to recovery in sheep with footrot. Journal of veterinary internal medicine / American College of Veterinary Internal Medicine 24: 420–425. 10.1111/j.1939-1676.2009.0450.x 20051002

[26] Hall JA, Sendek RL, Chinn RM, Bailey DP, Thonstad KN, et al (2011) Higher whole-blood selenium is associated with improved immune responses in footrot-affected sheep. Vet Res 42: 99. 21896161 10.1186/1297-9716-42-99 PMC3179948

[27] Raadsma HW, O'Meara TJ, Egerton JR, Lehrbach PR, Schwartzkoff CL (1994) Protective antibody titres and antigenic competition in multivalent Dichelobacter nodosus fimbrial vaccines using characterised rDNA antigens. Veterinary immunology and immunopathology 40: 253–274. 7909183 10.1016/0165-2427(94)90024-8

[28] Escayg AP, Hickford JGH, Bullock DW (1997) Association between alleles of the ovine major histocompatibility complex and resistance to footrot. Research in Veterinary Science 63: 283–287. 9491458 10.1016/s0034-5288(97)90035-7

[29] Egerton JR, Roberts DS (1971) Vaccination against Ovine Foot-Rot. Journal of Comparative Pathology 81: 179–&. 10.1016/0021-9975(71)90091-0 5091660

[30] Schwartzkoff CL, Egerton JR, Stewart DJ, Lehrbach PR, Elleman TC, et al (1993) The Effects of Antigenic-Competition on the Efficacy of Multivalent Footrot Vaccines. Australian Veterinary Journal 70: 123–126. 8098601 10.1111/j.1751-0813.1993.tb06101.x

[31] Hugejiletu H, Bobe G, Vorachek WR, Gorman ME, Mosher WD, et al (2013) Selenium supplementation alters gene expression profiles associated with innate immunity in whole-blood neutrophils of sheep. Biol Trace Elem Res 154: 28–44. 23754590 10.1007/s12011-013-9716-6

[32] NRC (2007) Nutrient Requirements of Small Ruminants: Sheep, Goats, Cervids, and New World Camelids. Washington, DC: National Academy Press.

[33] Bulgin MS, Lincoln SD, Lane VM, South PJ, Dahmen JJ, et al (1985) Evaluating an Ovine Foot-Rot Vaccine. Veterinary Medicine 80: 105–113.

[34] Winter AC (2009) Footrot control and eradication (elimination) strategies. Small Ruminant Research 86: 90–93.

[35] Wander RC, Hall JA, Gradin JL, Du SH, Jewell DE (1997) The ratio of dietary (n-6) to (n-3) fatty acids influences immune system function, eicosanoid metabolism, lipid peroxidation and vitamin E status in aged dogs. Journal of Nutrition 127: 1198–1205. 9187636 10.1093/jn/127.6.1198

[36] Brigelius-Flohe R, Kipp A (2009) Glutathione peroxidases in different stages of carcinogenesis. Biochim Biophys Acta 1790: 1555–1568. 19289149 10.1016/j.bbagen.2009.03.006

[37] Heirman I, Ginneberge D, Brigelius-Flohe R, Hendrickx N, Agostinis P, et al (2006) Blocking tumor cell eicosanoid synthesis by GP x 4 impedes tumor growth and malignancy. Free Radic Biol Med 40: 285–294. 16413410 10.1016/j.freeradbiomed.2005.08.033

[38] Kim KH, Gao Y, Walder K, Collier GR, Skelton J, et al (2007) SEPS1 protects RAW264.7 cells from pharmacological ER stress agent-induced apoptosis. Biochem Biophys Res Commun 354: 127–132. 17210132 10.1016/j.bbrc.2006.12.183 PMC1855283

[39] Curran JE, Jowett JB, Elliott KS, Gao Y, Gluschenko K, et al (2005) Genetic variation in selenoprotein S influences inflammatory response. Nat Genet 37: 1234–1241. 16227999 10.1038/ng1655

[40] Kipp AP, Banning A, van Schothorst EM, Meplan C, Coort SL, et al (2012) Marginal selenium deficiency down-regulates inflammation-related genes in splenic leukocytes of the mouse. The Journal of nutritional biochemistry 23: 1170–1177. 22137268 10.1016/j.jnutbio.2011.06.011

[41] Hall JA, Chinn RM, Vorachek WR, Gorman ME, Jewell DE (2010) Aged Beagle dogs have decreased neutrophil phagocytosis and neutrophil-related gene expression compared to younger dogs. Vet Immunol Immunopathol 137: 130–135. 20605222 10.1016/j.vetimm.2010.05.002

[42] Gomez CR, Nomellini V, Faunce DE, Kovacs EJ (2008) Innate immunity and aging. Experimental gerontology 43: 718–728. 18586079 10.1016/j.exger.2008.05.0168.05.016 PMC2564282

[43] Tiwary AK, Stegelmeier BL, Panter KE, James LF, Hall JO (2006) Comparative toxicosis of sodium selenite and selenomethionine in lambs. Journal of veterinary diagnostic investigation : official publication of the American Association of Veterinary Laboratory Diagnosticians, Inc 18: 61–70. 10.1177/104063870601800108 16566258

[44] Whanger PD (2002) Selenocompounds in plants and animals and their biological significance. J Am Coll Nutr 21: 223–232. 12074249 10.1080/07315724.2002.10719214

[45] Felix K, Gerstmeier S, Kyriakopoulos A, Howard OM, Dong HF, et al (2004) Selenium deficiency abrogates inflammation-dependent plasma cell tumors in mice. Cancer Res 64: 2910–2917. 15087411 10.1158/0008-5472.can-03-2672

[46] Novoselov SV, Calvisi DF, Labunskyy VM, Factor VM, Carlson BA, et al (2005) Selenoprotein deficiency and high levels of selenium compounds can effectively inhibit hepatocarcinogenesis in transgenic mice. Oncogene 24: 8003–8011. 16170372 10.1038/sj.onc.1208940

[47] Moustafa ME, Carlson BA, Anver MR, Bobe G, Zhong N, et al (2013) Selenium and selenoprotein deficiencies induce widespread pyogranuloma formation in mice, while high levels of dietary selenium decrease liver tumor size driven by TGFalpha. PLoS One 8: e57389. 23460847 10.1371/journal.pone.0057389 PMC3583866

[48] Hall JA, Bailey DP, Thonstad KN, Van Saun RJ (2009) Effect of parenteral selenium administration to sheep on prevalence and recovery from footrot. J Vet Intern Med 23: 352–358. 19192142 10.1111/j.1939-1676.2008.0253.x

[49] Sammalkorpi K, Valtonen V, Alfthan G, Aro A, Huttunen J (1988) Serum Selenium in Acute Infections. Infection 16: 222–224. 3182086 10.1007/BF01650756

[50] Kennan RM, Han X, Porter CJ, Rood JI (2011) The pathogenesis of ovine footrot. Vet Microbiol 153: 59–66. 21596496 10.1016/j.vetmic.2011.04.005

[51] Bennett GN, Hickford JG (2011) Ovine footrot: new approaches to an old disease. Vet Microbiol 148: 1–7. 20926208 10.1016/j.vetmic.2010.09.003

[52] Cederlof SE, Hansen T, Klaas IC, Angen O (2013) An evaluation of the ability of Dichelobacter nodosus to survive in soil. Acta Vet Scand 55: 4. 23343097 10.1186/1751-0147-55-4 PMC3561160

[53] Duncan JS, Grove-White D, Moks E, Carroll D, Oultram JW, et al (2012) Impact of footrot vaccination and antibiotic therapy on footrot and contagious ovine digital dermatitis. The Veterinary record 170: 462. 22266683 10.1136/vr.100363

[54] Woolaston RR (1993) Factors Affecting the Prevalence and Severity of Footrot in a Merino Flock Selected for Resistance to Haemonchus-Contortus. Australian Veterinary Journal 70: 365–369. 8257313 10.1111/j.1751-0813.1993.tb00809.x

[55] Kaler J, Daniels SL, Wright JL, Green LE (2010) Randomized clinical trial of long-acting oxytetracycline, foot trimming, and flunixine meglumine on time to recovery in sheep with footrot. J Vet Intern Med 24: 420–425. 20051002 10.1111/j.1939-1676.2009.0450.x

